# Identification of Prolyl isomerase Pin1 as a novel positive regulator of YAP/TAZ in breast cancer cells

**DOI:** 10.1038/s41598-019-42767-w

**Published:** 2019-04-23

**Authors:** Prem Khanal, Benjamin Yeung, Yulei Zhao, Xiaolong Yang

**Affiliations:** Department of Pathology and Molecular Medicine, Queen’s University, Kingston, Canada

**Keywords:** Molecular medicine, Breast cancer, Extracellular signalling molecules

## Abstract

The Hippo signalling pathway plays very important roles in tumorigenesis, metastasis, organ size control, and drug resistance. Although, it has been shown that the two major components of Hippo pathway, YAP and TAZ, play very crucial role in tumorigenesis and drug resistance, the exact molecular mechanisms are still unknown. Recently, we have shown that the prolyl isomerase Pin1 regulates the activity of Hippo pathway through interaction with Hippo component LATS kinase. Thus we asked if Pin1 is also able to interact with other Hippo pathway components. Therefore, in order to investigate whether Pin1 can interacts with other components of the Hippo pathway, we performed GST-pull down and co-immunoprecipitation (Co-IP) assays and have identified two Hippo components YAP and TAZ oncoproteins as novel binding partner of Pin1. We found that Pin1 interacts with YAP/TAZ in a phosphorylation-independent manner and WW domain of Pin1 is necessary for this interaction. Moreover, by using real time qRT-PCR, Cycloheximide chase, luciferase reporter, cell viability and soft agar assays, we have shown that Pin1 increases the tumorigenic and drug-resistant activity of YAP/TAZ through stabilization of YAP/TAZ at protein levels. Together, we have identified Pin1 as a novel positive regulator of YAP/TAZ in tumorigenesis and drug resistance of breast cancer cells. These findings will provide a significant contribution for targeting the Pin1-YAP/TAZ signaling for the successful treatment of tumorigenesis and drug resistance of breast and other cancers in the future.

## Introduction

Breast cancer is one of the major types of cancers diagnosed in women worldwide and accounts for 25% of all cancer cases and is responsible for 15% of cancer mortalities in women^1^. Although tremendous progress has been made toward our understanding of the molecular mechanism underlying breast cancer development, the successful cure for breast cancer is still a challenge due to cancer metastasis and drug resistance^2–6^. Therefore, there is an urgent need to identify novel targets or signalling pathway critical for the development of breast cancer and drug resistance. Pin1 is a Peptidyl-prolyl cis-trans isomerase (PPIase), consists of an N-terminal WW domain and a C-terminal PPIase domain. Usually, the WW domain of Pin1 binds to and isomerizes specific phosphorylated Serine/Threonine-Proline (S/T-P) motifs of its substrates, resulting in conformational changes of its substrate proteins which eventually lead to the regulation of many functions such as protein stability, tumorigenesis, phosphorylation status, cellular localization and other activities of its substrates^7–11^. Pin1 is overexpressed in many human cancers and drives numerous oncogenic processes including drug resistance, tumour development *in vitro* and *in vivo*, and poor clinical outcomes in human cancer patients^11–15^. Moreover, we and others have already shown that knockdown or inhibition of Pin1 is related with the decrease in tumorigenesis and increase in sensitivity of cancer cells to different anticancer drugs such as Tamoxifen and Trastuzumab^13,16–18^. Although many studies have been already carried out to elucidate the molecular mechanisms and different biomarkers which regulate the activity of Pin1 towards the cancer development and drug resistance, the downstream targets mediating Pin1 functions remain to be identified.

The transcriptional co-activators YAP and its paralog TAZ are the main downstream components of the Hippo pathway which are involved in cell proliferation, drug resistance and many other tumorigenic processes via transactivation of downstream genes such as CTGF, Cyr61, and BMP4 in the nucleus through transcription factor TEAD or Smad^19–28^. Moreover, it has been already shown that YAP and TAZ are involved in tumorigenesis of many cancers including breast cancer^29–31^. YAP and TAZ are also the major causes of resistance of different anticancer drugs such as Taxol and doxorubicin^23,32,33^. Currently many studies explain the detailed mechanisms for negative and positive regulation of YAZ/TAZ activity. For example, during negative regulation, the upstream components of Hippo signalling pathway MST1/2 kinases phosphorylate and activate S/T kinases LATS1/2, which in turn subsequently phosphorylate S127/S89 of YAP/TAZ preventing them from translocation to nucleus to activate transcription of downstream genes^25,34^. Moreover, serum deprivation, low mechanical stress, low glucose and nutrients, as well as diffusible signals that inhibit cell proliferation and metabolism are activators of the Hippo pathway core kinases and thus inhibit YAP/TAZ nuclear activity. On the other hand, low cell density, mitogenic signals, inflammation, and high nutrient uptake activate nuclear YAP and TAZ^35^. Recently, we showed that YAP and TAZ are positively regulated by PIK3CB to promote mammary tumorigenesis both *in vitro* and *in vivo*^36^. Although, there are numerous studies examining the negative regulators of YAP/TAZ activity, only very few positive regulators have been identified. In this study, we have identified Pin1 as a novel regulator of YAP and TAZ and have shown that Pin1 positively regulates YAP and TAZ activity towards tumorigenesis and Taxol resistance of human immortalized mammary epithelial MCF10A cells. Our studies identified Pin1 as a novel positive regulator of Hippo components YAP and TAZ. Future combination therapy which can target both Pin1 and YAP/TAZ might be a successful strategy for the treatment of drug resistance and tumorigenesis of breast cancer.

## Results

### Pin1 interacts with YAP *in vitro* and *in vivo*

**Figure 1:**
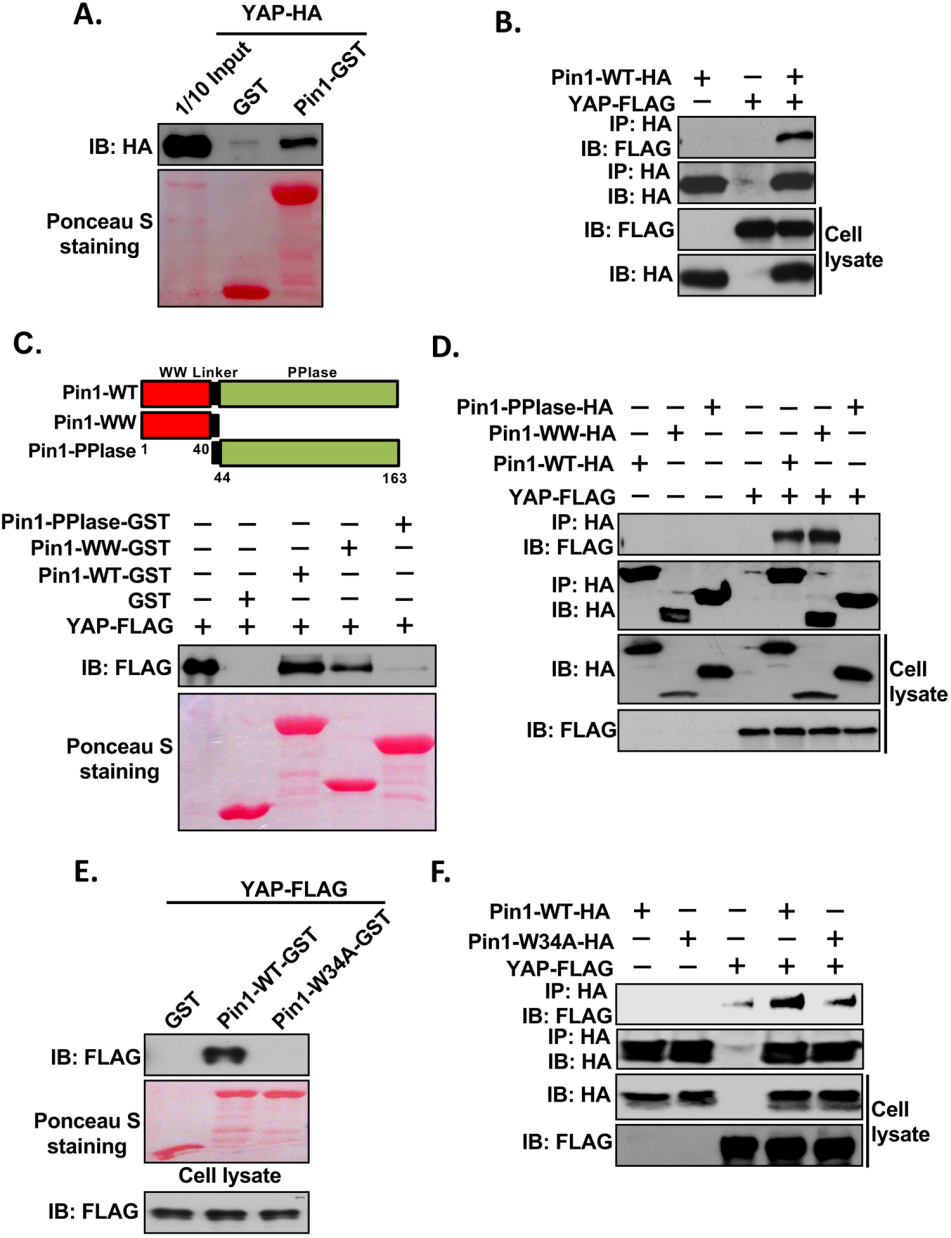
Interaction of Pin1 with YAP *in vitro* and *in vivo*. (**A**) Pin1 interacts with YAP *in vitro*. 200 µg of cell lysates from HA-tagged-YAP plasmid transfected HEK293 cells were precleared with 50% GSB beads overnight at 4 °C. After then, supernatants were mixed with 5 µg of GST or Pin1-GST and incubated for 2 hrs followed by addition of 20 μl of 50% GSB beads for another 1 hr. The beads were then washed, eluted by 2XSDS sample buffer and subjected to western blotting against anti-HA antibody. Ponceau-S staining shows the equal amount of fusion protein used for pull down. 1/10 input (20 µg) represents 1/10 of protein lysate used for pull down. (**B**) Pin1 interacts with YAP *in vivo*. HEK293 cells were transfected with HA-tagged-Pin1-WT or FLAG-tagged-YAP plasmids alone or together. The cells were harvested in 1%NP-40 lysis buffer. After checking the expression level of HA-tagged-Pin1-WT or FLAG-tagged-YAP, equal amount of cell lysates were subjected to co-immunoprecipitation assays using anti-HA antibody and immublotting analysis were performed using anti-FLAG or anti-HA antibody respectively. (**C**) Upper Panel, schematic diagram of full length (1–163) Pin1 (Pin1-WT), the WW domain (1–44) of Pin1 (Pin1-WW) and the PPIase domain (40–163) of Pin1 (Pin1-PPIase or Pin1-ΔWW). Bottom Panel, WW domain of Pin1 required for interaction of Pin1 with YAP *in vitro*. The lysates from FLAG-tagged-YAP transfected HEK293 cells were precleared with 50% GSB beads overnight at 4 °C. After then, supernatants were mixed with 5 µg of GST or Pin1-WT-GST, Pin1-WW-GST or Pin1-PPIase-GST separately and incubated for 2 hrs followed by addition of 20 μl of 50% GSB beads for another 1 hr. The beads were then washed, eluted by 2XSDS sample buffer and subjected to western blotting against anti-FLAG antibody. (**D**) WW domain of Pin1 is necessary for interaction of Pin1with YAP *in vivo*. HEK293 cells were transfected with HA-tagged-Pin1-WT, -WW, -PPIase or FLAG-tagged-YAP plasmids alone or together. The cells were harvested in 1%NP-40 lysis buffer. After checking the expression level of HA-tagged-Pin1-WT,-WW and -PPIase or FLAG-tagged-YAP, equal amount of cell lysates were subjected to co-immunoprecipitation assays using anti-HA antibody and immublotting analysis were performed using anti-FLAG or anti-HA antibody respectively. (**E**) Mutation at W34A at WW domain of Pin1 abolishes its interaction with YAP *in vitro*. GST pull down assay was carried out as mentioned in  (**A**) using the FLAG-YAP transfected HEK293 cell lysates and GST, Pin1-WT-GST or Pin1-W34A-GST. (**F**) Mutation at W34A at WW domain of Pin1 abolishes the interaction of Pin1 with YAP *in vivo*. HEK293 cells were transfected with HA-tagged-Pin1-WT, -W34A or FLAG-tagged-YAP plasmids alone or together. The cells were harvested in 1% NP-40 lysis buffer and co-immunoprecipitation was carried out as described in (**B**).

We have previously shown that Pin1 interacts with the two major components (LATS1 and LATS2) of the Hippo signalling pathway^37^. In order to further investigate whether the other components of Hippo signalling pathway also interact with Pin1, we first carried out the GST pull-down assays between Pin1 and YAP. For this, protein lysates extracted from HEK293 cells transfected with HA-tagged YAP2L expression vector and purified Pin1-GST fusion protein were used. Interestingly, we found that YAP interacts with Pin1 (Fig. 1A). Next, we used Co-IP assays to examine whether YAP can interact with Pin1 *in vivo*. FLAG-tagged YAP and HA-tagged Pin1 were transfected alone or together into HEK293 cells. The resulting cell lysates were immunoprecipitated with HA antibody. The results showed that YAP was found in the immune complex confirming its interaction with Pin1 (Fig. 1B). Since it has been already reported that Pin1 interacts with its substrates through its WW domain, we next examined if WW domain of Pin1 is also necessary for its interaction with YAP. When GST pull-down assays were carried out with cell lysates expressing FLAG-tagged YAP and different Pin1 GST fusion proteins (Pin1-WT, -WW and -PPIase domain) purified from bacteria, only the WT and WW domain of Pin1 was found to bind with YAP (Fig. 1C). This was further verified with Co-IP assays using lysates that were transfected with FLAG-tagged YAP and either HA-tagged Pin1-WT, -WW or -PPIase alone or together (Fig. 1D). As it has been already reported that a point mutation in the WW domain of Pin1 (Pin1-W34A) abolishes the interaction of Pin1 with its substrates^38^, we next examined whether it applies for Pin1-YAP interaction. GST pull down assay was performed using the cell lysates from YAP-FLAG-transfected HEK293 cells and GST, Pin1-WT-GST or Pin1-W34A-GST. As expected, we found that Pin1-W34A mutation abolished its interaction with YAP *in vitro* (Fig. 1E). This was further confirmed by Co-IP experiment using lysates that were transfected with YAP-FLAG and either Pin1-WT-HA, or -W34A-HA alone or together (Fig. 1F). In conclusion, these experiments indicate that Pin1 binds with YAP *in vitro* and *in vivo* through its WW domain.

### Phosphorylation-independent interaction of Pin1 and YAP

**Figure 2:**
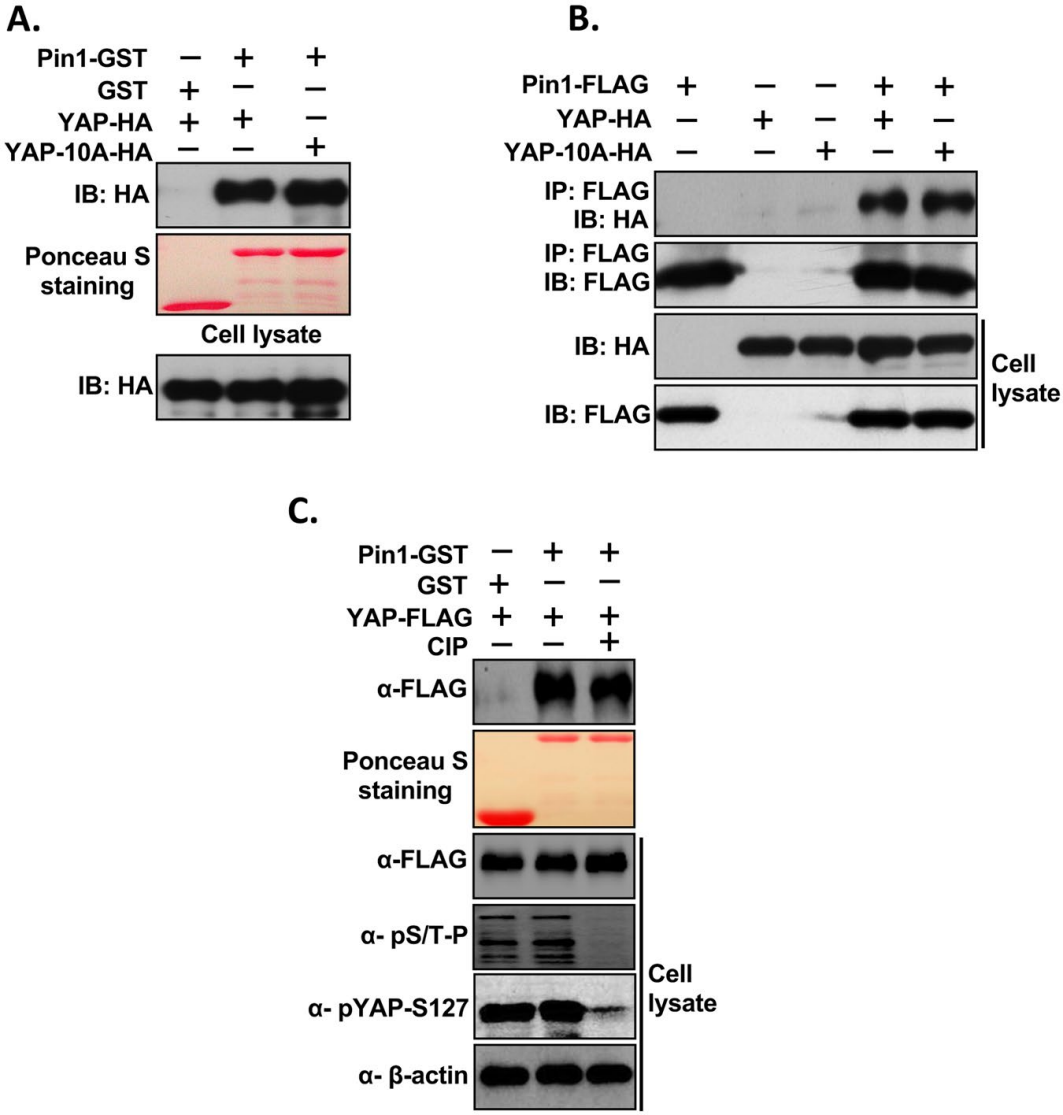
The interaction of Pin1 with YAP is independent of Phosphorylation of YAP. (**A**) Pin1 interacts with S/T-P mutants of YAP *in vitro*. All (10) Serine-or Threonine sites followed by Proline of YAP (S/T-P) were mutated to alanines (YAP-10A). 200 µg of cell lysates from HA-tagged-YAP-WT/YAP-10A plasmids transfected HEK293 cells were precleared with 50% GSB beads overnight at 4 °C and GST pull down assay was carried out as described in Method section using Pin1-GST. (**B**) Pin1 interacts with S/T-P mutants of YAP *in vivo*. Co-IP was carried out as described above. In brief, HA-tagged-YAP-10A was transfected alone or together with FLAG-tagged Pin1-WT into HEK293 cells and cells were harvested in 1% NP 40 lysis buffer after 48 hrs of transfection. After checking the expression equal amount of lysates were subjected to co-immunoprecipitation. (**C**) Inhibition of phosphorylation of YAP did not abolish the interaction of Pin1 with YAP. GST-pull down was carried out using the FLAG-tagged-YAP transfected HEK293 cell lysates treated or not treated with calf intestinal phosphatase (CIP) and Pin1-GST.

Since Pin1 interacts with specific phosphorylated serine or threonine residues that precede proline (pS/T-P) of its substrate^39^, we next examined whether the interaction of Pin1 and YAP is dependent on the phosphorylation of S/T-P motifs of YAP. For this, we first mutated all ten SP/TP sites of YAP2L (YAP-10A) and GST-pull assay was carried out using the lysates from HEK293 cells transfected with YAP-WT-HA or YAP-10A-HA and Pin1-GST. Surprisingly, we found that Pin1 still interacts with YAP-10A (Fig. 2A). Next, in order to confirmed this result, we performed Co-IP experiments using the lysates from HEK293 cells that were transfected with Pin1-FLAG or HA tagged-YAP-WT/-YAP-10A alone or in combination. This result also showed that Pin1 still interact with all ten SP/TP mutant of YAP (YAP-10A) suggesting that this interaction between Pin1 and YAP is independent of phosphorylation of Pin1’s substrate (Fig. 2B). Next, to further confirm whether this interaction of Pin1 with YAP is independent of phosphorylation, GST-pull down was carried out using FLAG-YAP-WT transfected HEK293 cell lysates treated with calf intestinal phosphatase (CIP) and Pin1-GST.The result showed that Pin1 still interact with YAP even after inhibition of YAP phosphorylation by CIP (Fig. 2C). All together these results suggest that the interaction of Pin1 with YAP is phosphorylation independent and it might be indirect interaction.

### Pin1 interacts with TAZ *in vitro* and *in vivo*

**Figure 3:**
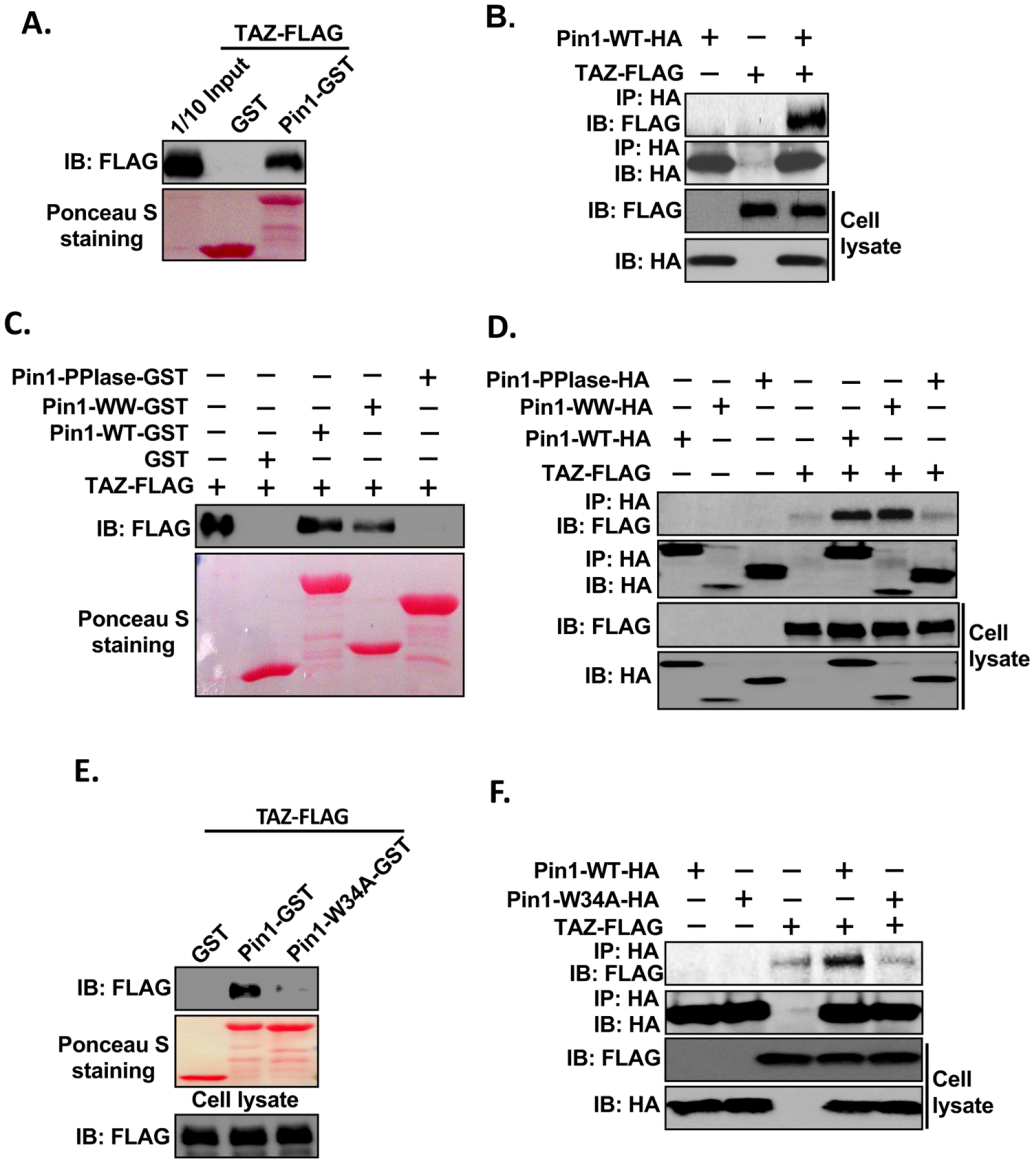
Pin1 interacts with TAZ *in vitro* and *in vivo*. (**A**) Pin1 interacts with TAZ *in vitro*. 200 µg of cell lysates from FLAG-tagged-TAZ plasmid transfected HEK293 cells were precleared with 50% GSB beads overnight at 4 °C. After then, supernatants were mixed with 5 µg of GST or Pin1-GST and incubated for 2 hrs followed by addition of 20 μl of 50% GSB beads for another 1 hr. The beads were then washed, eluted by 2XSDS sample buffer and subjected to western blotting against anti-FLAG antibody. Ponceau-S staining shows the equal amount of fusion protein used for pull down. 1/10 input (20 µg) represents 1/10 of protein lysate used for pull down. (**B**) Pin1 interacts with TAZ *in vivo*. HEK293 cells were transfected with HA-tagged-Pin1-WT or FLAG-tagged-TAZ plasmids alone or together. The cells were harvested in 1%NP-40 lysis buffer. After checking the expression level of HA-tagged-Pin1-WT or FLAG-tagged-TAZ, equal amount of cell lysates were subjected to co-immunoprecipitation assays using anti-HA antibody and immublotting analysis were performed using anti-FLAG or anti-HA antibody respectively. (**C**) WW domain of Pin1 interacts with TAZ *in vitro*. GST pull down assay was carried out as described above using the lysate from FLAG-tagged-TAZ transfected HEK293 cells and GST or Pin1-WT-GST, Pin1-WW-GST or Pin1-PPIase-GST separately (**D**) Pin1-WW domain is necessary for interaction of Pin1with TAZ *in vivo*. Co-IP was carried out as described above by using the cell lysate from FLAG-tagged-TAZ or HA-Pin1-WT,- WW or -Pin1-PPIsae alone or together transfected HEK293 cells. Anti-HA antibody was used for immunoprecipitation and immublotting analysis were performed using anti-FLAG or anti-HA antibody respectively. (**E**) Pin1-W34A mutant abolish its interaction with TAZ *in vitro*, GST pull down assay was carried out as mentioned in Fig. 1A using the FLAG-TAZ transfected HEK293 cell lysates and GST, Pin1-WT-GST or Pin1-W34A-GST. (**F**) Mutation at W34A at WW domain of Pin1 abolish the interaction of Pin1 with TAZ *in vivo*. HEK293 cells were transfected with HA-tagged-Pin1-WT, -W34A or FLAG-tagged-TAZ plasmids alone or together. The cells were harvested in 1%NP-40 lysis buffer and co-immunoprecipitation was carried out as described above.

Since TAZ, a paralog of YAP, shows around 60% protein sequence similarity and similar characteristics towards cell proliferation, cell transformation, tumorigenesis, and both of them are key downstream effectors of the Hippo pathway^40–42^, we were interested to determine whether TAZ can also interact with Pin1. To that end, we first performed GST pull-down assays as we did for YAP using the cell lysates from TAZ-FLAG transfected HEK293 cells and Pin1-GST. Interestingly, the result showed that TAZ also interacts with Pin1 *in vitro* (Fig. 3A). Furthermore, *in vivo* interaction of TAZ with Pin1 was confirmed by Co-IP by transfecting HEK293 cells with Pin1-HA or TAZ-FLAG alone or together (Fig. 3B). Next, we mapped the domain of Pin1 which is responsible for interaction with TAZ using GST pull-down assay. TAZ-FLAG was transfected into HEK293 cells and total cell lysates were subjected to pull-down assay using GST fusion protein containing different fragments of Pin1 as shown in Fig. 1C. As in the case of YAP, the result showed that only WT and WW, but not PPIase domain of Pin1, could interact with TAZ (Fig. 3C). This result was confirmed by Co-IP experiment by transfecting HEK293 cells with TAZ-FLAG and/or HA-tagged Pin1-WT, -WW and -PPIase alone or together (Fig. 3D). We next investigated whether or not mutation of Tryptophan (W) at position 34 in the WW domain of Pin1 to alanine (Pin1-W34A) abolishes the interaction of Pin1 with TAZ. Both GST pull-down (Fig. 3E) and Co-IP (Fig. 3F) assays showed that Pin1-W34A mutation completely abolishes the interaction of Pin1 with TAZ *in vitro* and *in vivo*. All together these results indicate that WW domain of Pin1 is important for its interaction with TAZ.

### Pin1 interacts with TAZ in a Phosphorylation-independent manner

**Figure 4:**
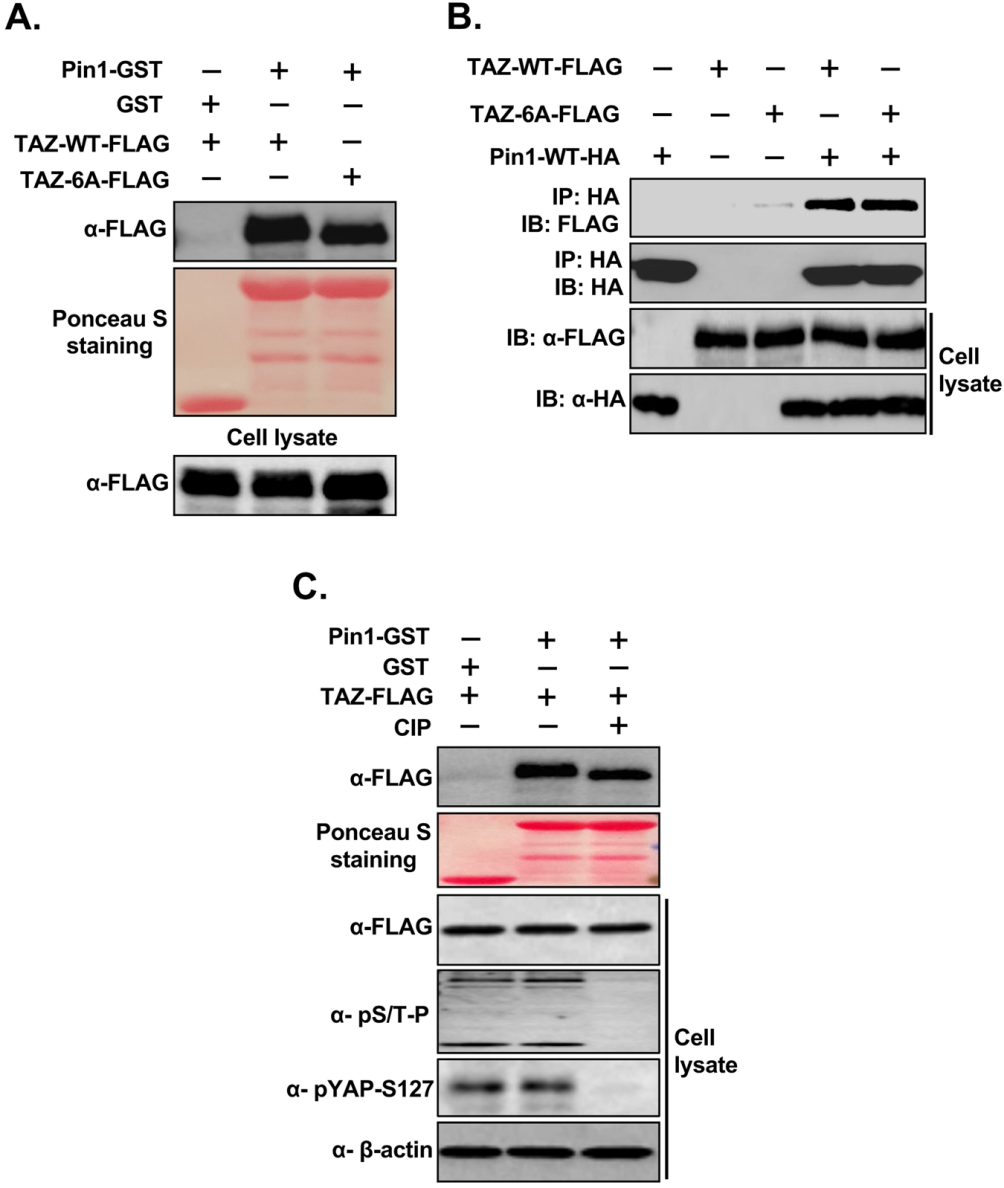
Phosphorylation independent interaction of Pin1 with TAZ *in vitro* and *in vivo*. (**A**) Pin1 interacts with S/T-P mutant of TAZ *in vitro*, all (six) S or T sites followed by Proline of TAZ (S/T-P) were mutated to alanines (TAZ-6A). 200 µg of cell lysates from FLAG-tagged-TAZ-WT/TAZ-6A plasmids transfected HEK293 cells were precleared with 50% GSB beads overnight at 4 °C and GST pull down assay was carried out as described using GST or Pin1-GST. (**B**) Pin1 interacts with S/T-P mutant of TAZ *in vivo*. Co-IP was carried out as described above. In brief, FLAG-tagged-TAZ-WT/TAZ-6A were transfected alone or together with HA-tagged Pin1-WT into HEK293 cells and cells were harvested in 1% NP 40 lysis buffer after 48 hrs of transfection. After checking the expression, equal amount of lysates were subjected to co-immunoprecipitation. (**C**) Inhibition of phosphorylation of TAZ did not abolish the interaction of Pin1 with TAZ, GST-pull down was carried out using the FLAG-tagged-TAZ transfected HEK293 cell lysates treated or not treated with calf intestinal phosphatase (CIP) and Pin1-GST.

Next, we determined whether the interaction of Pin1 with TAZ is also phosphorylation independent. For this, we mutated all six SP/TP sites of TAZ (TAZ-6A) and performed GST pull-down assays using the cell lysate from HEK293 cells transfected with FLAG-tagged TAZ-WT or-TAZ-6A and Pin1-GST. Similar to YAP, we found that Pin1 still interact with TAZ even after mutation of all its SP/TP sites (Fig. 4A). This result was further confirmed by Co-IP experiment using the cell lysate from HEK293 cells transfected with HA-tagged Pin1 or FLAG-tagged TAZ-WT/TAZ6A alone or together (Fig. 4B). These results indicate that Pin1 interacts with TAZ in phosphorylation-independent manner. In order to further confirm this phosphorylation-independent interaction of Pin1 with TAZ, we next performed the GST pull down assay using the lysate from CIP treated TAZ-FLAG transfected HEK293 cell lysates and Pin1-GST. The result showed that treatment of CIP causes downward shift of the TAZ-FLAG but did not abolish the interaction of Pin1 with TAZ (Fig. 4C), suggesting that the interaction of Pin1 with TAZ is phospho-independent and it might be indirect interaction.

**Figure 5:**
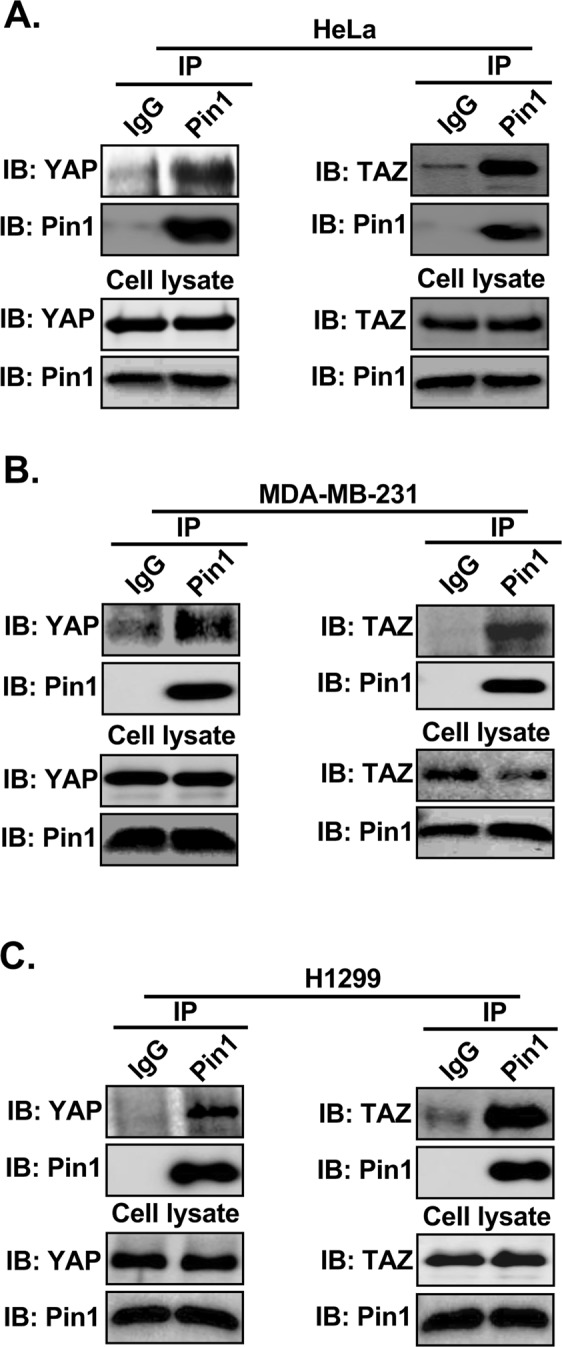
Pin1 interacts with YAP/TAZ endogenously. (**A**–**C**) Pin1 interacts with YAP/TAZ *in vivo.* 3 mg of cell lysate from different cell lines HeLa (A), MDA-MB-231(B) and H1299 (C) were subjected to co-immunoprecipitation assays using anti-rabbit IgG or anti-Pin1 antibody separately and immublotting analysis were performed using anti-YAP/TAZ or anti-Pin1 antibody respectively.

After confirming the phosphorylation independent interaction of Pin1 with YAP/TAZ, we next examined whether the interaction of Pin1 and YAP/TAZ was detectable at the endogenous level. HeLa cervical, MDA-MB-231 breast and H1299 lung cancer cells were harvested in 1% NP40 lysis buffer and subjected to immunoprecipitation with anti-IgG or anti-Pin1 antibody, separately and blotted with anti-YAP/TAZ or Pin1 antibodies respectively. Interestingly, we found that Pin1 interacts with YAP/TAZ in all three cell lines used (Fig. 5A–C) indicating the presence of endogenous interaction of Pin1 with YAP/TAZ in physiological condition.

### Pin1 increases the stability of YAP/TAZ in breast cancer cells

**Figure 6:**
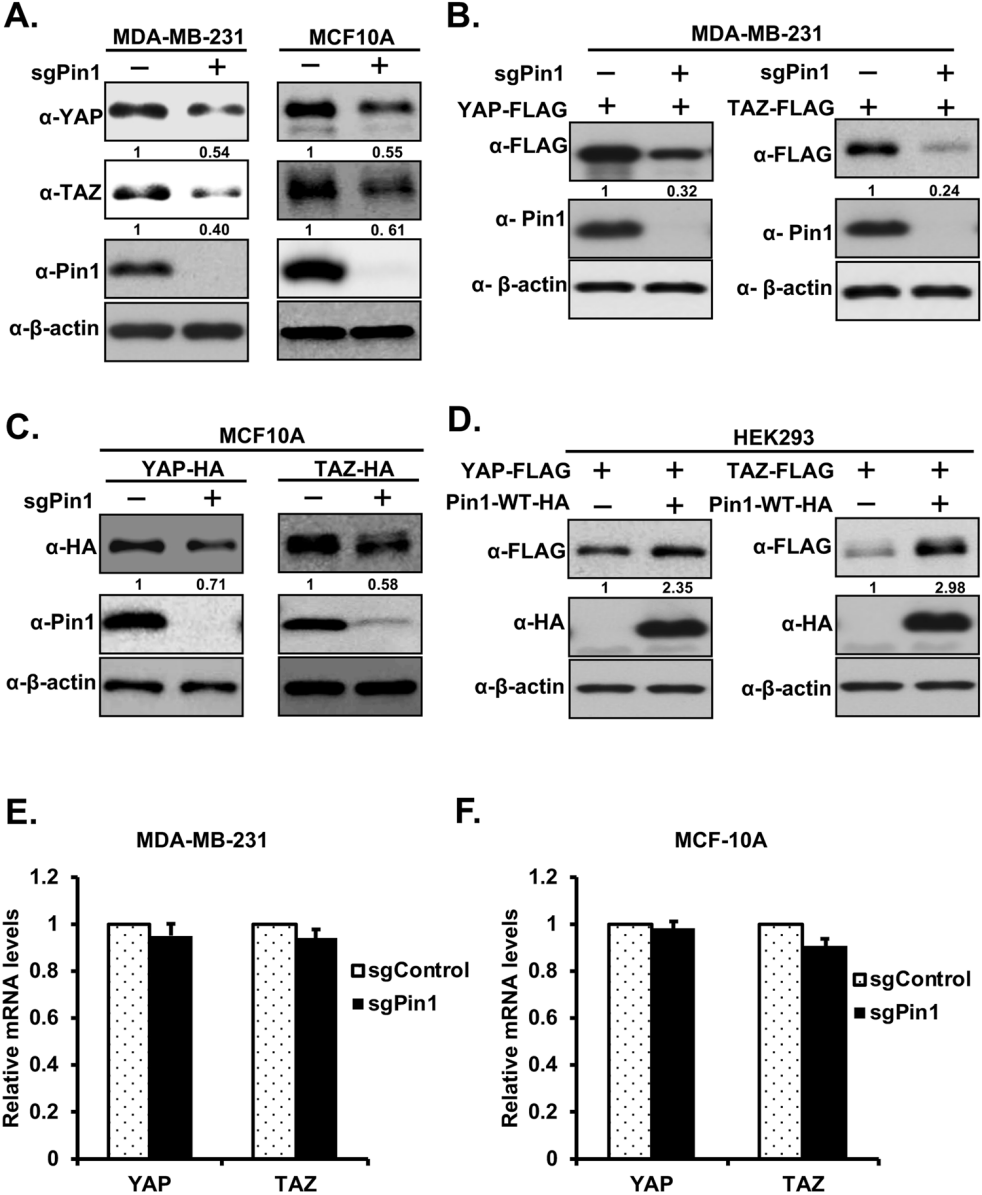
Pin1 increases the expression of YAP/TAZ proteins. (**A**) Knockout of Pin1 decreases the expression of endogenous YAP/TAZ proteins. Pin1 was knockout in MDA-MB-231(left panel) and MCF10A (right panel) using sgRNA-Pin1 as described in experimental procedure section. The cell lysates from sgRNA-control or sgRNA-Pin1 infected MDA-MB-231/MCF10A stable cell lines were subjected to western blotting and blotted with respective antibodies as shown in figure. (**B**) Knockout of Pin1 decreases the ectopic expression of YAP/TAZ proteins, equal amount of FLAG-tagged YAP/TAZ were transfected separately in to sgRNA-control or sgRNA-Pin1 MDA-MB-231 stable cell lines. After 48 hrs of transfection cells were harvested in RIPA lysis buffer and western blotting was carried out using the antibodies as indicated. (**C**) Knockout of Pin1 decreases the expression of YAP/TAZ proteins in WPI-HA-YAP/TAZ-MCF10A stable cell lines. The cell lysates from control or Pin1 knockout WPI-HA-YAP/TAZ-MCF-10A cell lines were separated by western blotting using the respective antibodies as indicated in figure. (**D**) Overexpression of Pin1 increases ectopic expression of YAP/TAZ proteins in HEK293 cells. Cells were transfected with FLAG-YAP/TAZ expression vector alone or together with HA-tagged-Pin1-WT plasmid. 48 hrs after transfection cells were harvested in RIPA lysis buffer and immublotting was carried our using the corresponding antibodies as shown in figure. (**E**,**F**) Knockout of Pin1 did not affect the total mRNA level of YAP/TAZ. Total mRNA was extracted from sgRNA-control or sgRNA-Pin1 MCF-10A stable cell lines. Real-time PCR (RT-PCR) was performed with primers targeting YAP (**E**) and TAZ (**F**). Ribosomal RNA was used as an internal control.

In order to investigate the effect of Pin1 on expression of YAP/TAZ proteins, we first knocked out Pin1 in MDA-MB-231 breast cancer cells using CRISPR-Cas9, followed by immunoblotting to confirm gene knockout. We found that knockout of Pin1 decreases the levels of endogenous YAP and TAZ proteins (Fig. 6A, left panel and Supplementary Fig. 1A, left panel). To ensure that this decreased level of endogenous YAP/TAZ proteins in Pin1 knockout cells is not cell line specific, we knocked out Pin1 in MCF10A mammary cells as before and checked the level of endogenous YAP/TAZ proteins by western blotting. The result is consistent with those obtained in MDA-MB231 cells (Fig. 6A, right panel and Supplementary Fig. 1A, right panel). Addback of PAM-mutated Pin1-WT but not Pin1-WW-mutant (Pin1-ΔWW) into Pin1 knockout MDA-MB-231 and MCF10A cell lines restores endogenous YAP/TAZ expression (Supplementary Fig. 2A,B), further supporting that Pin1 increases the stability of YAP/TAZ.

Moreover, to investigate whether Pin1 regulates the ectopic expression of YAP/TAZ proteins, we overexpressed FLAG-tagged YAP/TAZ separately in MDA-MB-231-sgControl or -sgPin1 stable cell lines. After 48 hrs of transfection, cells were harvested in RIPA lysis buffer and western blotting was carried out against anti-FLAG, -Pin1 and -β-actin antibodies respectively. The results showed that knockout of Pin1 decreases the expression level of ectopic YAP/TAZ proteins (Fig. 6B and Supplementary Fig. 1B). We next knocked out Pin1 in MCF10A cell lines stably overexpressing YAP/TAZ (MCF10A-YAP/TAZ-HA) and found that knockout of Pin1 in these cell lines decreases the overexpressed YAP/TAZ proteins levels (Fig. 6C and Supplementary Fig. 1C). Furthermore, in order to further confirm the role of Pin1 in regulation of YAP/TAZ proteins, HEK293 cells were overexpressed with FLAG-tagged YAP/TAZ alone or together with Pin1-WT-HA. The results showed that the ectopic expressions of YAP/TAZ proteins were increased by Pin1 overexpression (Fig. 6D). Next, to explore if enhanced levels of YAP/TAZ by Pin1 are due to transcription regulation, we examined the mRNA levels of *YAP/TAZ* in Pin1 knockout MDA-MB-231 and MCF10A cells. Interestingly, the quantitative real time PCR (qRT-PCR) results showed that the *YAP/TAZ* mRNA levels were not affected by knockout of Pin1 in MDA-MB-231 and MCF-10A cells (Fig. 6E,F), suggesting that Pin1 affects the stability of YAP and TAZ at protein levels.

### Pin1 increases the stability of YAP/TAZ proteins

**Figure 7:**
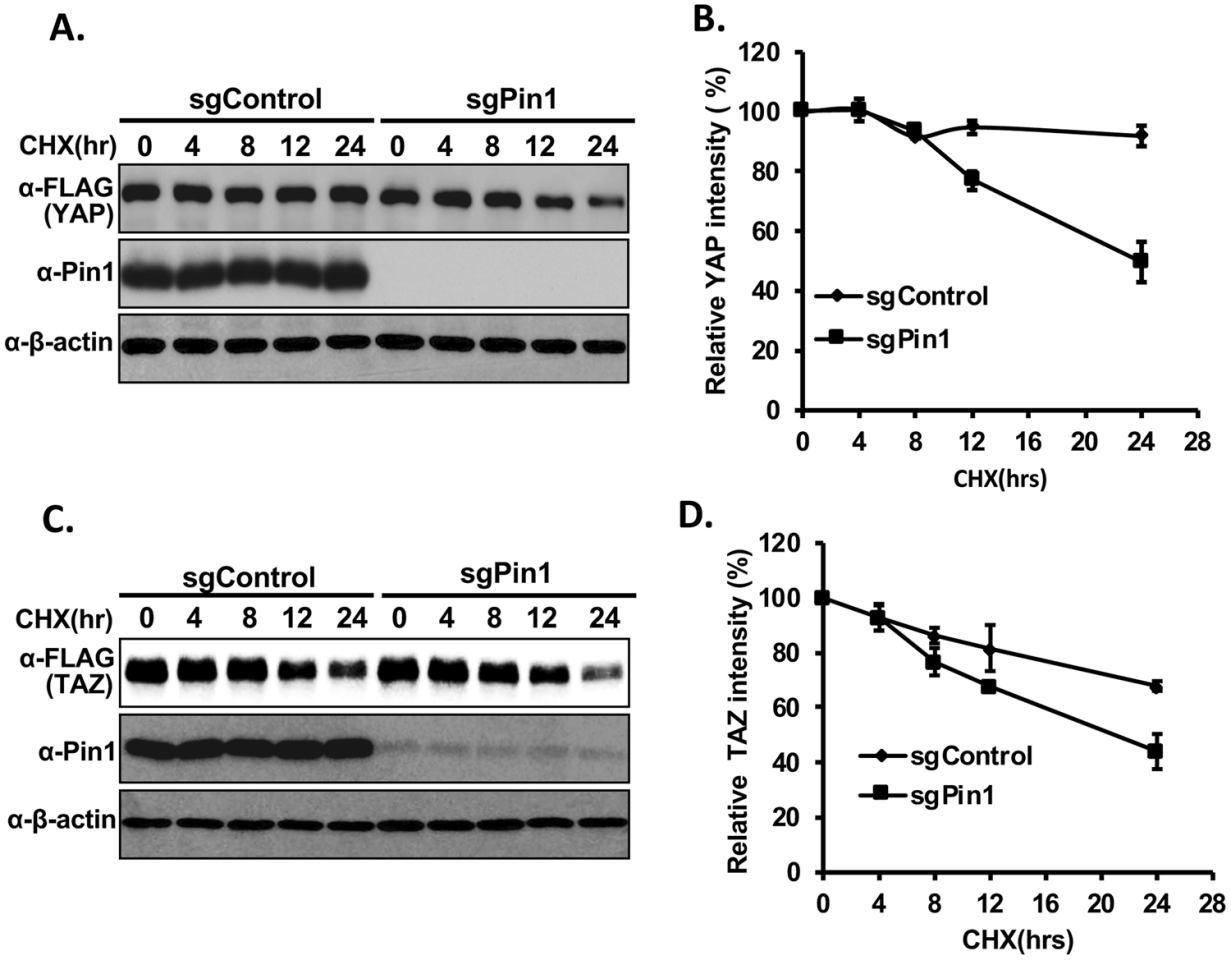
Pin1 increases the stability of YAP/TAZ proteins. (**A**–**D**) YAP and TAZ are degraded more in Pin1 knockout cells. Equal amount of FLAG-tagged YAP (A, B) or FLAG-tagged TAZ (**C**,**D**) was transfected into sgControl or sgPin1 stable HEK293 cell lines separately. 24 hrs after transfection, cells were treated with 100 μg/ml cycloheximide and harvested at different time as indicated in figure. Immublot was carried out using the respective antibodies as shown.

To check whether Pin1 regulates the stability of YAP/TAZ proteins, we performed a cycloheximide chase experiment. For this, YAP/TAZ-FLAG plasmids were transfected into control or Pin1 knockout HEK293 stable cell lines separately, followed by cycloheximide treatment. The results showed that inhibition of translation led to increased YAP/TAZ protein degradation in Pin1 knockout cell as compared to control cells (Fig. 7A–D) confirming our above findings that Pin1 regulates YAP/TAZ protein stability rather than transcription. Next, we checked whether the ubiquitin-mediated proteolytic pathway is responsible for YAP/TAZ degradation by comparing protein stability in the absence or presence of proteasome inhibitor MG132 in control or Pin1 knockout HEK293 cells. The results indicate that treatment of MG132 significantly inhibited the degradation of YAP/TAZ proteins in the absence of Pin1, suggesting that Pin1 increases the stability of YAP/TAZ proteins by inhibiting the ubiquitin-mediated degradation (Supplementary Fig. 3A,B).

### Pin1 increases the activity of YAP/TAZ to induce CTGF and 3TP-lux promoter activity

**Figure 8:**
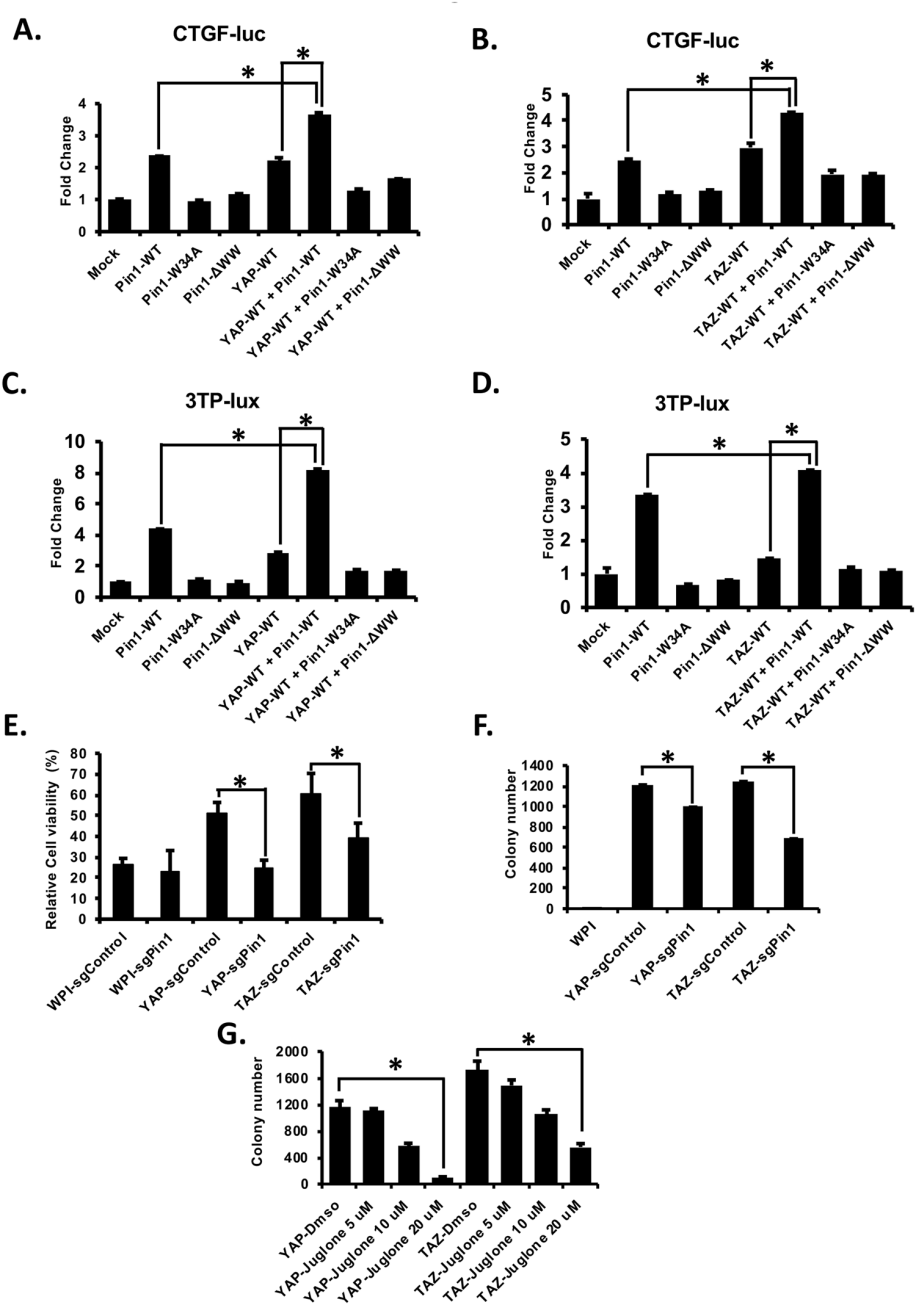
Pin1 enhanced the YAP/TAZ induced *CTGF*/*3TP-lux* promoter activity and oncogenic activity of YAP/TAZ. (**A**,**B**) Pin1 increases the activity of YAP/TAZ towards CTGF promoter activity. 100 ng of CTGF reporter was transfected into each well of 12 well plates along with 200 ng of each plasmid as indicated into HEK293 cells (total 500 ng/well). 48 hrs after transfection cells were harvested and luciferase assays were carried out as described in experimental sections. The firefly luciferase activity was measured in the cell lysates and normalized against the Renilla luciferase activity. (**C**,**D**) Pin1 co-operate with YAP/TAZ to induce 3TP-lux reporter activity. HEK-293 cells were transfected with the 100 ng/well of 3TP-lux–luc together with 200 ng/well of different plasmids as indicated in figure. 48 hrs after transfection lucifease assay was carried out as described above. All the luciferase assays were carried out in biological triplicates. *p < 0.05 (*t* test). (**E**) Deletion of Pin1 decreases the YAP/TAZ induced Taxol resistance in MCF10A cells. 1 × 10^4^ cells/well of control or Pin1 knockout MCF10A-WPI,-YAP, or-TAZ stable cell lines were plated in 96 well plate. After 24 hrs of plating cells were treated with 50 nM for Taxol for 48 hrs and cell viability was measured as described in experimental sections. (**F**) Knockout of Pin1 decreases the YAP/TAZ induced cell transformation activity in MCF10A cells, soft agar assay was performed as descried in experimental sections. In brief 3000 cells/well of control or Pin1knockout MCF10A-YAP/TAZ stable cell lines were plated for soft agar in 6-well plates (triplicate). (**G**) Inhibition of Pin1 decreases the YAP/TAZ induced colony formation of MCF10A cells as revealed by soft agar assay, 3000 cells/well of MCF-10A-YAP/TAZ stable cell lines were plated for soft agar in 6-well plates (triplicate) and cells were treated with Dmso or 5 µM, 10 µM and 20 µM of Pin1 inhibitor Juglone. The media was refreshed at every 2–3 days and colonies were stained and counted after 2–3 weeks. The average colony numbers were calculated and the colonies from 3 separate experiments were photographed. Bars represent means ± S.D. “*” represent significant difference (*P < *0.05) in *t*-test.

Next, in order to explore the role of Pin1 in YAP/TAZ activity, we choose two well-known promoters (CTGF and 3TP-lux) which were activated by YAP/TAZ^26,27,43^ and performed the luciferase assay. HEK293 cells were transfected with CTGF or 3TP-lux- reporters along with different expression plasmids alone or together as shown in figure. We found that YAP/TAZ-induced CTGF (Fig. 8A,B) and 3TP-lux (Fig. 8C,D) promoter activities were enhanced by wild type Pin1 (Pin1-WT) but not Pin1-WW mutants (Pin1-ΔWW or Pin1W34A). Moreover, we have also shown that Pin1 isomerase defective mutant Pin1-K63A could not co-operate with YAP/TAZ to induce 3TP-lux-promoter activity (Supplementary Fig. 4), suggesting the involvement of both WW and PPIase domain of Pin1 to enhance YAP/TAZ oncogenic activity. Altogether, these results suggest that WW domain of Pin1 interacts with YAP/TAZ and positively regulates their activity. Moreover, we have also shown that all serine-threonine/Proline (S/T-P) mutants of YAP (YAP-10A) and TAZ (TAZ-6A) still acts like YAP-WT and TAZ-WT to induce the CTGF and 3-TP-lux promoter activities in the absence or presence of Pin1 (Supplementary Fig. 5), supporting our finding that the interaction of Pin1 with YAP/TAZ is independent of phosphorylation of YAP/TAZ.

### Loss of Pin1 decreases the activity of YAP/TAZ toward Taxol resistance and cell transformation of MCF10A cells

Previous studies have already shown that YAP and TAZ are involved in Taxol resistance of mammary cells^23,33^. Therefore, to confirm that Pin1 regulates YAP/TAZ function, we next tested whether loss of Pin1 reduces YAP/TAZ activity toward Taxol resistance of MCF-10A breast epithelial cells. Control or Pin1 knockout MCF10A-WPI (vector), -YAP or -TAZ stably overexpressed cell lines were treated with 50 nM of Taxol separately for 48 hrs. Interestingly, the results showed that knockout of Pin1 decreases the cell viability as compared to their corresponding control cells, suggesting that Pin1 might cooperate with YAP/TAZ to induce Taxol resistance of breast cancer cells (Fig. 8E). To further confirm that Pin1 co-operates with YAP/TAZ, we performed soft agar assays in control or Pin1 knockout MCF-10A-YAP/TAZ stable cell lines. As expected, the colony forming ability of YAP/TAZ was inhibited by loss of Pin1 in YAP/TAZ stably overexpressed MCF10A cell lines (Fig. 8F). This result was further confirmed by inhibition of Pin1 by its inhibitor Juglone. Soft agar assays were carried out as described in experimental methods using YAP or TAZ stably overexpressed MCF10A cell lines with or without Pin1 inhibitor Juglone. Interestingly, we found that inhibition of Pin1 by Juglone decreases the colony forming capacity of YAP/TAZ in MCF-10A cells (Fig. 8G). All together these results suggest that Pin1 positively regulates YAP/TAZ and work together to induce the Taxol resistance and tumorigenesis of breast cancer.

## Discussion

YAP and TAZ are the major downstream components of the Hippo signalling pathway and are involved in different tumorigenic^24,44–47^, and pro-apoptotic functions^48,49^. It has been shown that the activities of YAP/TAZ are negatively regulated by the upstream components MST1/2 and LATS1/2 in the Hippo signalling pathway which involved the phosphorylation of LATS1/2 by MST^50^ and subsequently the phosphorylated and activated LATS1/2 phosphorylates YAP and TAZ^20,25,34,51^. The phosphorylated YAP/TAZ is then inactivated through sequestration in the cytoplasm, proteosomal degradation, and nuclear exclusion^25,51,52^. It has also been shown that other than LATS 1/2, YAP and TAZ can be negatively regulated by non-Hippo components, such as CDK1^33,53^, AMOT^54^, α-catenin^55^, PTPN14^56^ and c-Abl^57^. Although, there are numerous amounts of studies which characterize negative regulators of YAP/TAZ, the positive regulators of YAP/TAZ are still not well studied and need to be investigated more. Our study identified Pin1 as a novel positive regulator of YAP/TAZ (Fig. 8), which increases the levels or stabilities of YAP/TAZ (Figs 6 and 7). Moreover, we found that WW domain of Pin1 interacts with YAP/TAZ (Figs 1 and 3) and knockout/inhibition of Pin1 decreases the oncogenic functions of YAP/TAZ (Fig. 8).

Pin1 is a peptidyl-prolyl cis/trans isomerase which interacts with many phosphorylated proteins leading to the changes in conformation of its substrate and these Pin1 induced conformational changes regulates the different functions of its substrate proteins including protein stability, enzyme activity, protein-protein interaction, subcellular localization and transcriptional activity^8,38,39,58–63^. Moreover, Pin1 regulates different cellular processes, including cell cycle progression, cell growth, cell migration, cell transformation, apoptosis, and cellular stress responses through regulation of large numbers of phosphorylated proteins^64^ leading to the different human diseases, including cancer^60,65^, Alzheimer’s disease^66^, and Parkinson disease^59^. Although, it has been shown that Pin1 regulates different cellular signalling pathways such as NF-kappaB signaling pathway^59^, insulin signalling pathway^67,68^, EGF signalling pathway^17^, Notch signalling pathway^69^, MAPK signalling pathway^13^ and SMAD signalling pathway^70^, its role in Hippo signalling pathway has not been previously explored. Here, for the first time, we have shown that Pin1 interacts with and increases the expression and stability of two major components (YAP/TAZ) of the Hippo signalling pathway (Figs 1, 3, 6 and 7). In addition, we found that WW domain of Pin1 interacts with YAP/TAZ *in vitro* and *in vivo* and this interaction is independent of phosphorylation of YAP/TAZ (Figs 1–5). Although, it has been reported that WW domain of Pin1 interacts with its substrate phosphorylated at S/T-P residues^38,39^, our results showed that WW domain of Pin1 interact with YAP/TAZ in a phosphorylation independent manner (Figs 2 and 4). In addition, purified Pin1-GST was unable to interact with purified YAP-GST in an *in vitro* binding assay (data not shown), indicating that Pin1-YAP interaction might be indirect. Since it has been reported that WW domain of YAP/TAZ interact with the PPXY motifs of its substrate^34^, so in order to check the possibility whether WW domain of YAP/TAZ interact with PPXY motif containing proteins which can bridge the interaction of Pin1 with YAP/TAZ, we performed Co-IP between the WW domain mutant of YAP/TAZ with Pin1 and found that Pin1 still interact with WW mutant of YAP/TAZ (Supplementary Fig. 6) excluding the possible involvement of PPXY motif proteins as a bridging proteins for Pin1-YAP/TAZ interaction. Nonetheless, it has been also reported that WW domain of Pin1 could interact with proteins via the residues other than pS/T-P^71^, which supports our findings that the interaction of WW domain of Pin1 with YAP/TAZ is phosphorylation-independent. It is possible that Pin1 indirectly interacts with YAP/TAZ through their commonly interacting proteins AP-1 or Smads^70,72–77^ as like as the interaction of Pin1 with α- synuclein^59^. Therefore, further identification and characterization of protein(s) mediating Pin1-YAP/TAZ interaction will be very helpful in elucidating the mechanism by which Pin1 regulates YAP/TAZ stability and function.

YAP and TAZ are involved in Taxol resistance of mammary cells^23,33^. Also, there are enormous amount of studies which showed that Pin1 is involved in multidrug resistance including Tamoxifen, Trastuzumab and Taxol^13,14,16–18,63^. However, there is no link between YAP/TAZ and Pin1 in regulation of drug sensibility. Our study provides evidence that inhibition of Pin1 decreases the Taxol resistance induced by YAP/TAZ overexpression in MCF10A mammary cells (Fig. 8E), indicating that Pin1 co-operates with YAP/TAZ to induce drug resistance in many cancer cells. Pin1 is overexpressed and activated in different kinds of cancer including breast, lung, gastric, melanoma, prostrate, ovary and cervical cancers and its overexpression correlates with poor clinical outcome in human cancer patient^62,65,78–81^. Pin1 overexpression also causes centrosome amplification, chromosome instability and tumor development *in vitro* and *in vivo*^8,82^. Likewise YAP/TAZ also involved in many tumorigenic processes. Although, Pin1 and YAP/TAZ had been shown to be involved in different oncogenic processes, the exact molecular mechanisms and regulators about how these proteins regulate oncogenic process are still need to be investigated. Our studies found that Pin1 enhanced the tumorigenic activity of YAP/TAZ (Fig. 8) suggesting that Pin1 is critical for YAP/TAZ induced tumorigenicity. At the same time, it might be possible that YAP/TAZ also synergize with Pin1 to enhance the tumorigenic or drug resistant activities of Pin1. Hence the detailed understanding about how Pin1-YAP/TAZ regulates each other will provide a novel strategy for the successful treatment of drug resistance and tumorigenecity of cancers. Moreover, since Pin1^83,84^ and YAP/TAZ^85–87^ were reported to involve in cancer stem cell signalling pathways, there is possibility that these genes co-operate with each other to regulate many cancer stem cell regulators and induce their self-renewal and tumorigenic properties leading to amplification of many oncogenic pathways responsible for development of cancer. Hence, there is a possibility that targeting Pin1-YAP/TAZ signalling could be a potential anti-cancer target. Therefore, it will be very interesting to further explore how Pin1 regulates YAP/TAZ or vice versa in various biological functions.

In summary, this study identified Pin1 as a novel positive regulator of two major downstream components (YAP and TAZ) of the Hippo signalling pathway and has shown that Pin-YAP/TAZ interaction increases the activity and stability of YAP and TAZ leading to the drug resistance and tumorigenecity of breast cancer cells. Further exploration about this signalling in the development of cancer and drug resistance and targeting of these proteins will have significant implication for successful treatment of cancer in the future.

## Experimental Procedures

### Plasmids construction, site-directed mutagenesis and transfection

Plasmids construction and site-directed mutagenesis were performed as described previously^34^. Full length cDNAs of human YAP2L (accession number NM_001130145.2, TAZ (accession number NM_001168278.2), human Pin1 (accession number NM_006221.3), WW and PPIase domain mutants of Pin1, YAP/TAZ-WW mutants and different S/T mutants of YAP2L or TAZ were subcloned into pcDNA3.1-hygro-3xFLAG, pcDNA3-HA, pGEX4T-1, HA-tagged WPI lentiviral vectors, respectively. Transfections of plasmids into cells were carried out by using PolyJet^TM^ (SignaGen) according to the manufacturer’s protocol.

### Cell culture

Dulbecco’s modified eagle medium (DMEM), Roswell Park Memorial Institute-1640 (RPMI-1640) (1X), and fetal bovine serum (FBS) were purchased from Invitrogen (Carlsbad, CA). HEK293, HEK293T, MDA-MB-231, H1299 (non-small cell lung carcinoma cell line), HeLa (cervical cancer cells) and MCF10A cells were purchased from ATCC. HEK293 (human embryonic kidney cells), HeLa and MDA-MB-231 (triple-negative breast cancer cells) cells were cultured in Dulbecco’s Modified Eagle’s Medium (DMEM; Sigma, #D6429) supplemented with 10% fetal bovine serum and 1% penicillin/streptomycin (P/S) (Invitrogen). H1299 cells were maintained in RPMI-1640 supplemented with 10% FBS and 1% P/S. MCF10A (human immortalized mammary epithelial cells) were cultured in DMEM/Nutrient Mixture F12 Ham (Sigma-Aldrich) supplemented with 5% horse serum (HS) (Invitrogen), 20 ng/mL hEGF, 500 ng/mL hydrocortisone, 10 mg/mL insulin, 2.5 mmol/L L-glutamine, 100 ng/mL cholera toxin, and 1% P/S with regular subculture at every 3–4 days with 1:10 ratio. Cells were cultured at 37 °C in humidified air containing 5% CO_2._

### Antibodies and reagents

Anti-FLAG-M2 and β-actin antibodies were from Sigma Aldrich. Antibodies against Pin1, YAP and HA-F7 were purchased from Santa Cruz Biotechnology (Santa Cruz, CA). TAZ antibody was from BD Transduction Laboratories. Phospho-YAP-S127 antibody was from Cell Signaling Technology. An antibody against phospho-S/T (MPM2) was from Millipore. Restriction enzymes for cloning were purchased from NEB (New England Bio Lab). Glutathione Sepharose bead was obtained from GE Healthcare. Protein G/A-Agarose was purchased from Roche Diagnostics.

### Lentivirus production, infection and establishment of stable cell lines

Lentivirus production and concentration were carried out as described before^34,88^. To make YAP/TAZ stable MCF-10A cell lines, 2 × 10^5^ cells were plated into each well of a 6-well plate. After 24 hrs of plating, cells were infected with WPI, WPI-HA-YAP-WT, and WPI-HA-TAZ-WT lentivirus respectively using 8 µg/ml of Polybrene in each well. The expression of respective genes was confirmed by western blot using anti-HA antibody. To knockout Pin1 in MCF10A-WPI, MCF-10A-WPI-HA-YAP and -TAZ cells, sgRNAs targeting Pin1 were cloned into lentiCRISPRv1 vector (Addgene) and were used for producing lentivirus and resulting viruses were infected into the cells as described above. 24 hrs after infection, cells were selected with 1 µg/mL Puromycin. The stable cell lines were either collected for protein analysis or functional assays. Pin1-WT and Pin1-ΔWW CRISPR-Cas9–resistant addback constructs were made by mutating the CRISPR-targeting PAM sequence in the Pin1 cDNA by overlapping PCR and were cloned into WPI vector and used to make corresponding lentiviruses and stable cell lines.

### GST fusion protein production and pull-down assays

GST fusion proteins were produced and purified as described previously^89^. For GST-pull down assays, 200 µg of respective protein lysates were pre-cleared overnight at 4 °C with 20 µL Glutathione Sepharose 4B beads. Next day, 5 µg of appropriate GST or GST fusion proteins were added to the corresponding supernatants and incubated at 4 °C for more 2 hr. 20 µL of 50% GSB was then added and further incubated for 1 hr. After this, beads were washed four times with lysis buffer (50 mM Tris-HCl pH 7.4, 150 mM NaCl, 1 mM EDTA and 1.0% Nonidet P-40) and 2xSDS sample buffer was added to beads and boiled for 10 mins and centrifuged. The supernatants were run on SDS-PAGE and blotted with respective antibodies.

### Immunoblotting, co-immunoprecipitation (Co-IP) and cycloheximide treatment

Immunoblotting and Co-IP was carried out as described previously^34^. In brief, cells grown to 70% to 80% confluency were harvested in RIPA/1%-NP40 lysis buffer. Protein samples were subjected to SDS-PAGE and immunoblotted with respective antibodies using standard protocol. For co-immunoprecipitation, cells expressing different genes were harvested in 1% NP-40 lysis buffer. After checking the expression of different proteins, equal amounts of proteins were precleared overnight at 4 °C using Protein-A/G-agarose. Then the supernatant were subjected to immunoprecipitation using anti-HA-F7/FLAG-M2 antibody for 2 hr at 4 °C. After 2 hr, 20 μl of protein- A/G-agarose was added for another 1 hr and beads were washed 4 times with 150 mM-NaCl-1% NP-40 lysis buffer. Then 20 μl of 2xSDS sample buffer was added to beads and boiled for 10 mins and centrifuged. The supernatants were run in SDS-PAGE and blotted with respective antibodies.

For cycloheximide treatment, equal amount of FLAG-tagged YAP or FLAG-tagged TAZ was transfected in to sgControl or sgPin1 stable HEK293 cell lines separately and 24 hrs after transfection, cells were treated with 100 μg/ml cycloheximide and harvested. Protein levels of YAP/TAZ-FLAG were examined by western blot analysis. The experiments were repeated two to three times.

### RNA extraction and qRT-PCR

To extract total RNA, cells were cultured until 70–80% confluency and total RNA was extracted by RNAzol®RT reagent (Molecular Research Center, Inc., Cincinnati, OH, USA) according to the manufacturer’s protocol. Real time PCR (qRT-PCR) analysis was performed as described before^34^. Data from qRT-PCR were statistically analyzed using unpaired t-tests, and *P* values < 0.05 were considered significant.

### Luciferase assay

2 × 10^5^ HEK293 cells/well were plated in 12 well plates, triplicate for each group. After 24 hrs of plating cells were transfected with 100 ng/well of CTGF-luc or 3TP-lux-luc alone or together with other plasmids using Polyjet (SignaGen). 10 ng/well of Renilla luciferase vector (pRL-TK) was cotransfected as an internal transfection control. After 48 hrs of transfection, luciferase activity was measured with the Dual Luciferase Reporter Assay System (Promega) and Turner Biosystems 20/20 luminometer.

### Cell viability assay

1 × 10^4^ cells per well were plated in 96 well plate. 24 hrs after plating cells were treated with Taxol at 50 nM or DMSO as control for 2 days. Then cell viability was measured through Cell Titer-Glo (Promega) and relative viability was further calculated based on the measured values of control (DMSO) and those treated with drugs. Result was presented as mean ± SD (n = 3). “*” represents the differences are significant, with P < 0.05.

### Anchorage-independent cell transformation assay (Soft agar assay)

Soft agar assay was performed as described before^34^. Briefly, triplicates of different MCF-10A stable cells (3 × 10^3^) were mixed with complete growth media containing 0.4% agarose and then plated over 0.8% agarose in each well of 6-well plates. After 24 hr, 1 ml of complete growth medium was added to each well. Culture media was refreshed at every 2–3 days. The cultures were maintained at 37 °C in a 5% CO_2_ incubator for 15 days and colonies were stained with 0.005% crystal violet in 20% methanol. Pictures were taken by using Bio-Rad Gel Doc System (Bio-Rad, Mississauga, Canada) and colonies were quantified by colony count program in Quantity One software. Data from soft agar assays were statistically analyzed using unpaired t-tests, and *P* values < 0.05 were considered significant.

## Supplementary information

Supplementary information
