# Supplementary material for: Identification of Prolyl isomerase Pin1 as a novel positive regulator of YAP/TAZ in breast cancer cells
Source: Sci Rep. 2019 Apr 23;9:6394. doi: 10.1038/s41598-019-42767-w (PMC6478839; doi:10.1038/s41598-019-42767-w)

Identification of Prolyl isomerase Pin1 as a novel positive regulator of YAP/TAZ in breast cancer cells

Prem Khanal[1, 2], Benjamin Yeung[1, 2], Yulei Zhao[2], and Xiaolong Yang[2]*

[1]PK and BY contributed equally to this work

[2]Department of Pathology and Molecular Medicine, Queen's University, Canada

Running title: Pin1 positively regulates YAP and TAZ

*To whom correspondence should be addressed: Xiaolong Yang, Ph.D., Richardson Lab 201 D, 88 Stuart Street, Kingston, ON K7L 3N6, Canada; Tel: 613 533-6000 Ext.75998, Fax: 1-613-533-2970. E-mail: yangx@queensu.ca

Keywords: Hippo pathway, YAP, TAZ, Pin1, soft agar assay, GST-pull down, cell-viability, Breast cancer, drug resistance

---

Supplementary Figures

Supplementary Figure 1. Densitometric analysis of YAP and TAZ western blots used in Fig. 6A, 6B and 6C. .

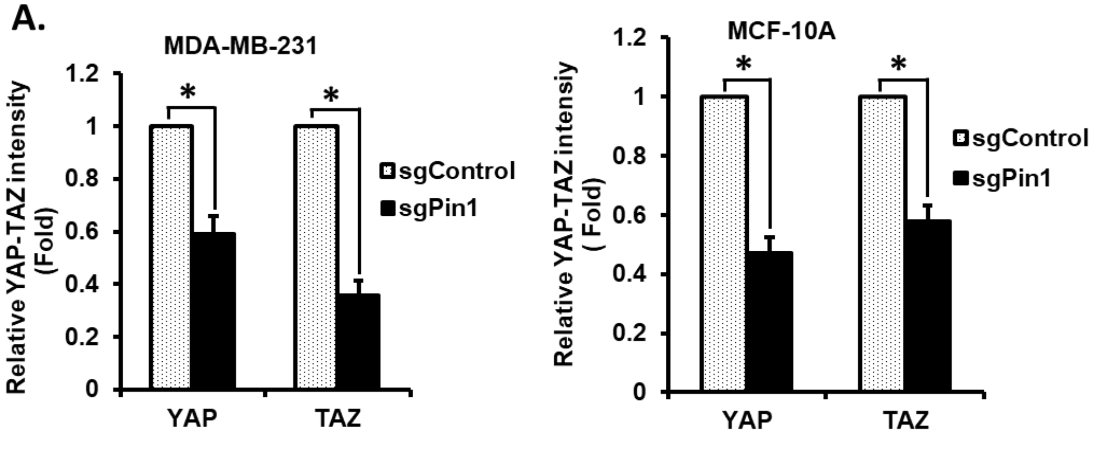

B.

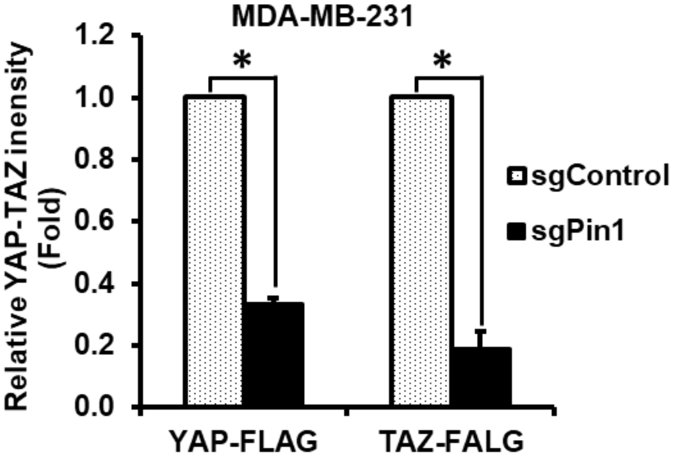

C.

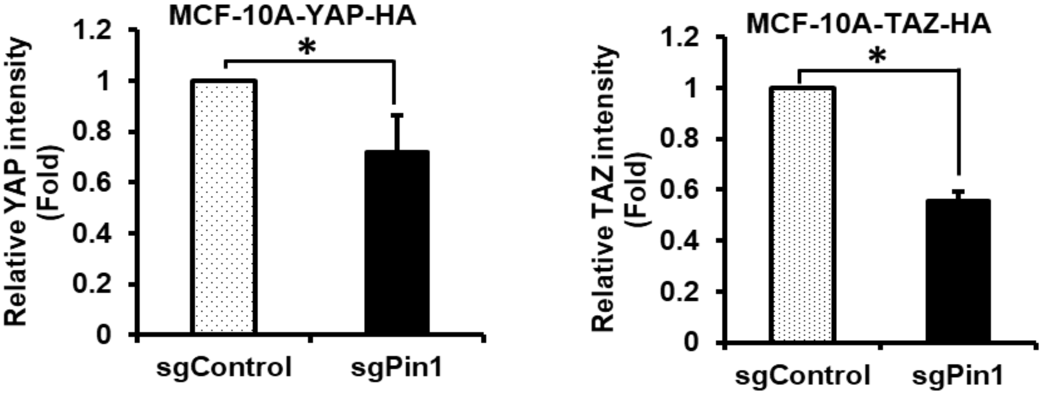

Supplementary Figure 2. Addback of PAM-mutated Pin1-WT but not Pin1-WW-mutant (Pin1-ΔWW) into Pin1 knockout-MDA-MB-231 (A) and -MCF-10A (B) cell lines restores endogenous YAP/TAZ expression.

A.

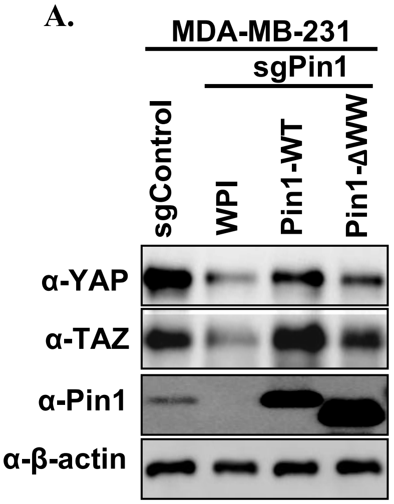

B.

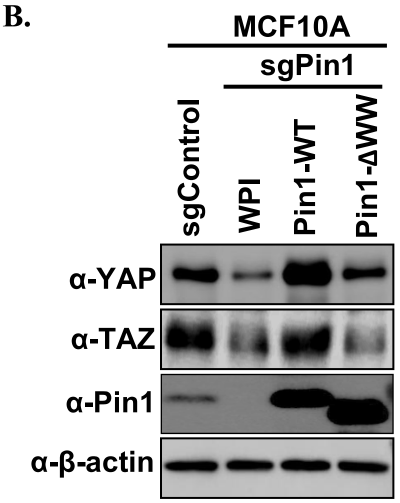

Supplementary Figure 3. Knockout of Pin1 decreases the stability of YAP/TAZ Protein. (A and B) FLAG-tagged YAP (A) and TAZ (B) were transfected in to sgRNA-control or sgRNA-Pin1 stable HEK293 cells separately and 24 hr after transfection cells were either treated or not treated with 20 µM of MG132 for 24 hrs. The cells were harvested in RIPA lysis buffer and immublotted.

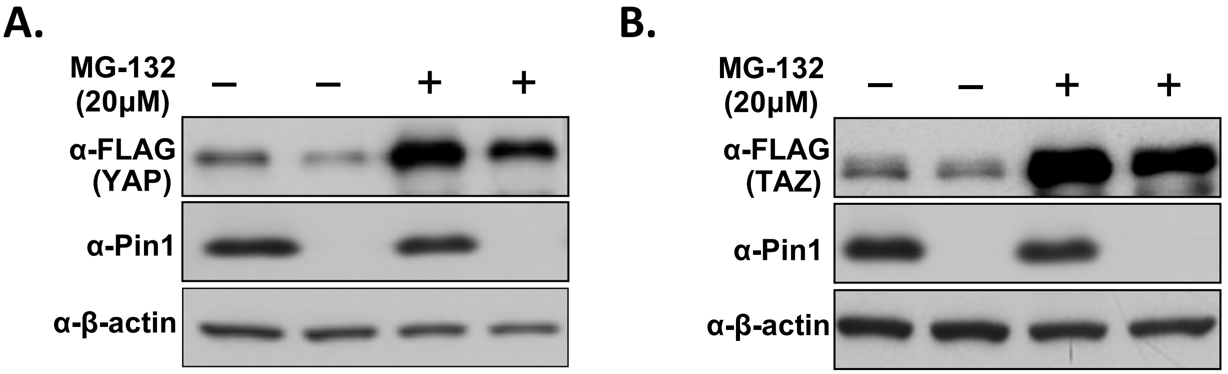

Supplementary Figure 4. Isomerase-defective Pin1 mutant, Pin1-K63A couldn't enhanced YAP/TAZ induced *3TP-lux* promoter activity. (A and B) Isomerase-defective Pin1 mutant, Pin1-K63A couldn't enhanced YAP (A) and TAZ (B) induced *3TP-lux* promoter activity.

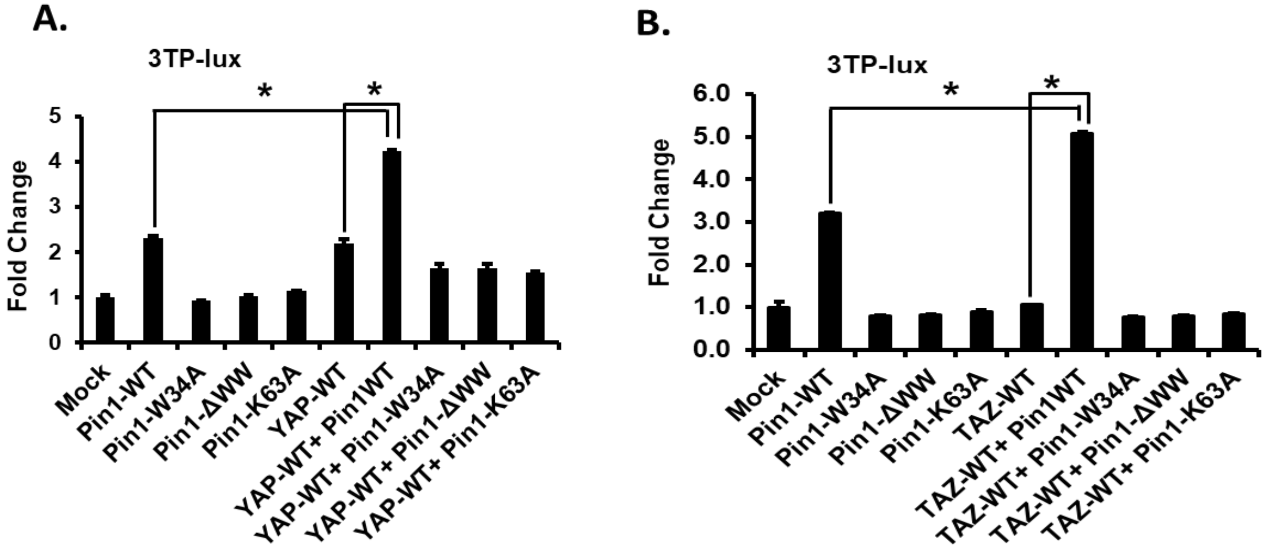

Supplementary Figure 5. Mutation of all ten S/T-P sites of YAP to Alanine (YAP-10A) or all six S/T-P sites of TAZ to Alanine(TAZ-6A) didn't abolish the Pin1 enhanced YAP/TAP induced CTGF/3TP-lux activity. (A and B) Mutation of all ten S/T-P sites of YAP (YAP-10A) (A) and all six S/T-P sites of TAZ (TAZ-6A) (B) did not inhibit the Pin1-WT enhanced YAP/TAZ induced CTGF promoter activity. (C and D) Mutation of all ten S/T-P sites of YAP (YAP-10A) (C) and all six S/T-P sites of TAZ (TAZ-6A) (D) did not inhibit the Pin1-WT enhanced YAP/TAZ induced 3TP-*lux* promoter activity.

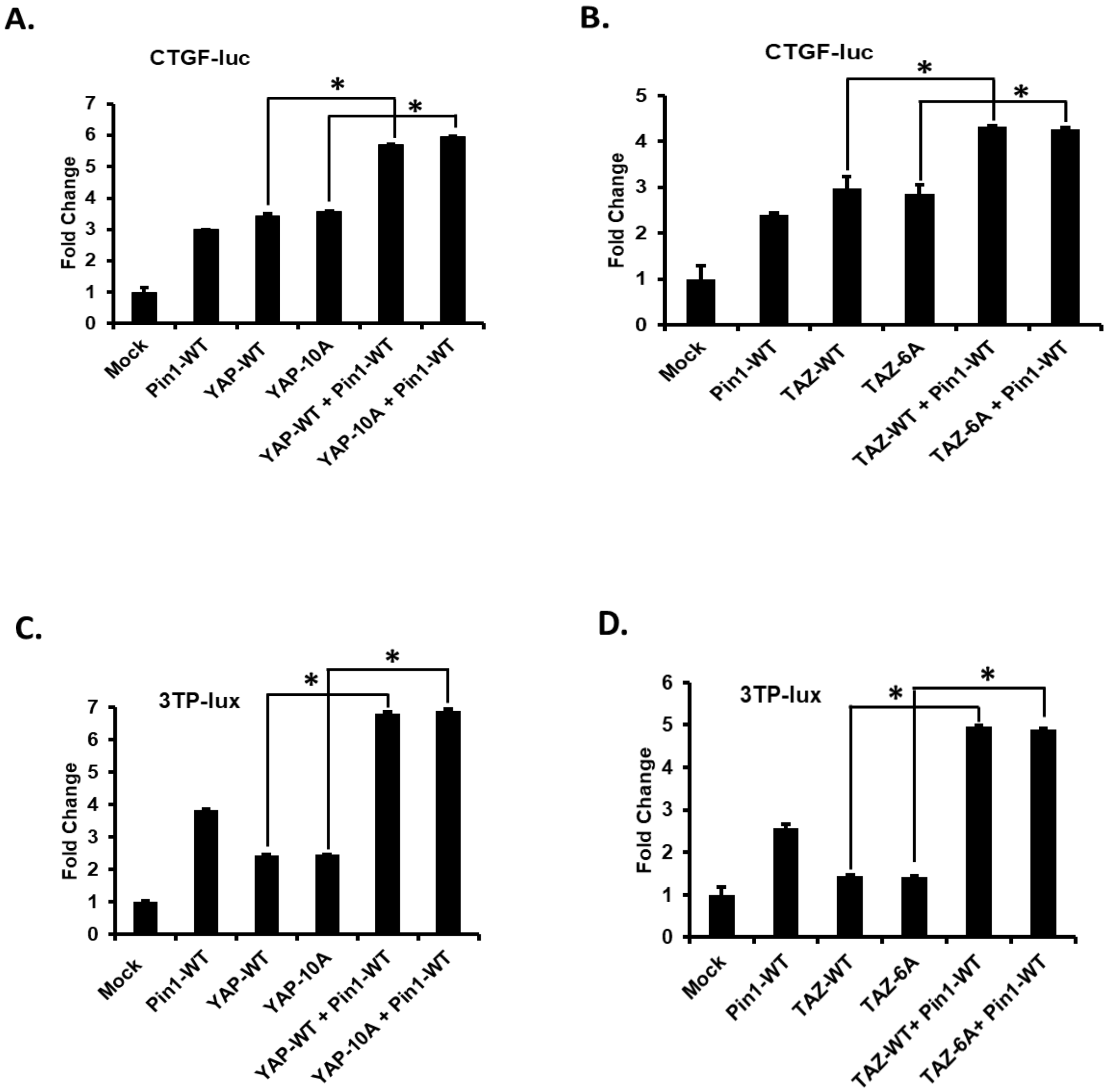

Supplementary Figure 6. Mutation of WW domain of YAP/TAZ did not abolish the interaction of Pin1 with Yap/TAZ. (A and B) Mutation of WW domain of YAP (YAP-WWm) (A) or TAZ(TAZ-WWm) (B) did not abolish the interaction of YAP/TAZ with Pin1.

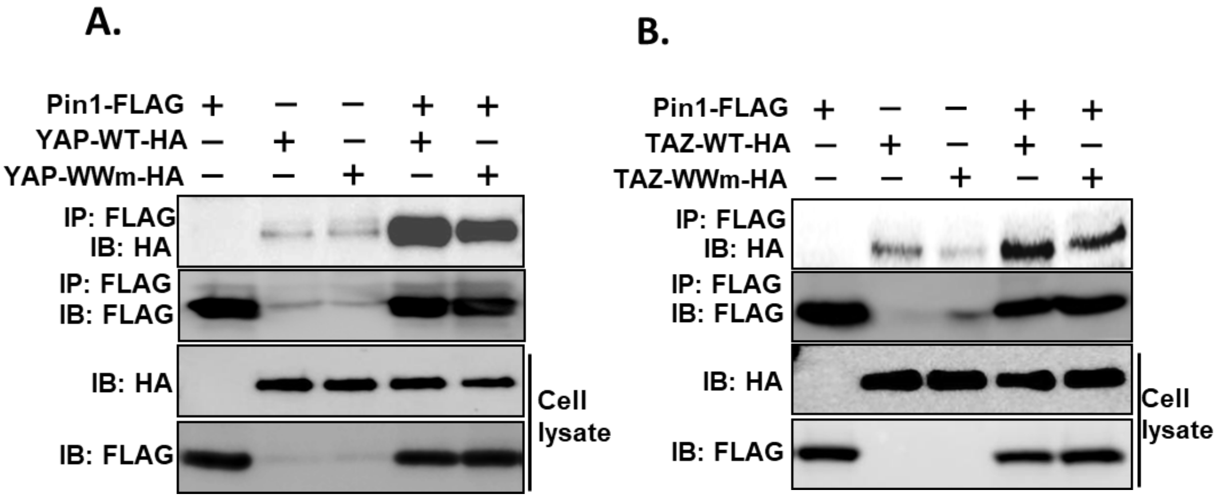

Supplementary Figure 7. (A) Full blots of crop blots used in Fig.1A, the parts of blots used in figure are highlighted with in square. (B) Original blots used in Fig.1B, the parts of blots used in figure are highlighted with in square. (C) Full blots used to make Fig. 1C. (D) Original blots used in Fig. 1D, the parts of blots used in figure are highlighted with in square. For IP: HA-Pin1 and IB: FLAG-YAP different blots with long and short exposure are shown. (E) Original blots used in Fig. 1E, the parts of blots used in figure are highlighted with in square. (F) Original blots used in Fig.1F, the parts of blots used in figure are highlighted with in square. For all western blots used in manuscript, membranes were either developed by using X-Ray film or Images were taken using Amersham Imager from GE-Health care.

(A)

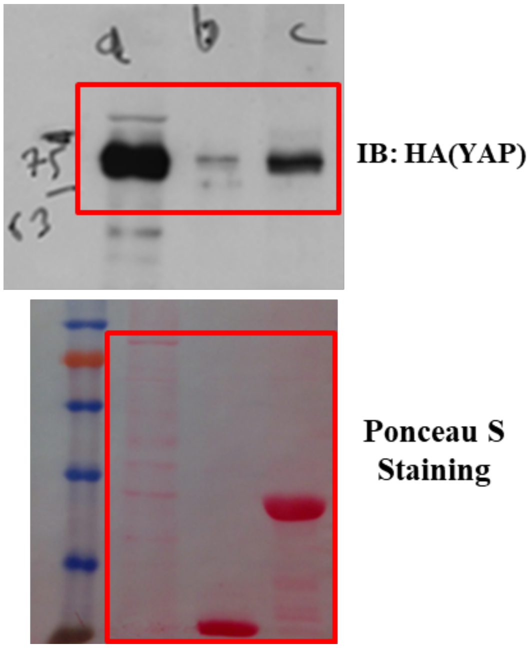
IB: HA(YAP)
Ponceau S Staining

(B)

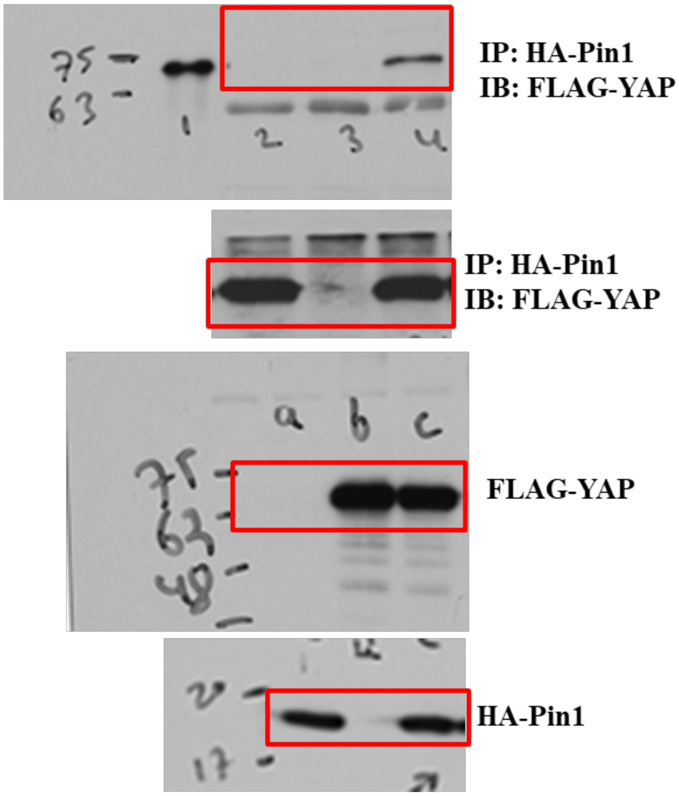
IP: HA-Pin1
IB: FLAG-YAP
IP: HA-Pin1
IB: FLAG-YAP
FLAG-YAP
HA-Pin1

(C)

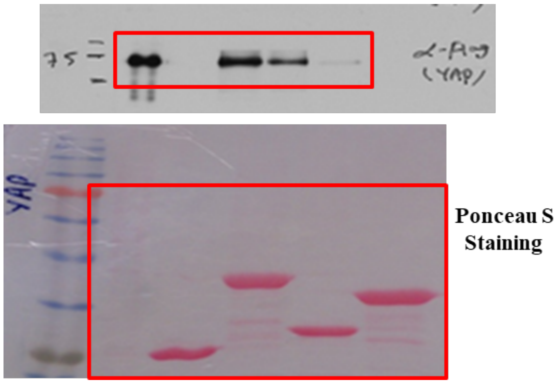
Ponceau S Staining

(D)

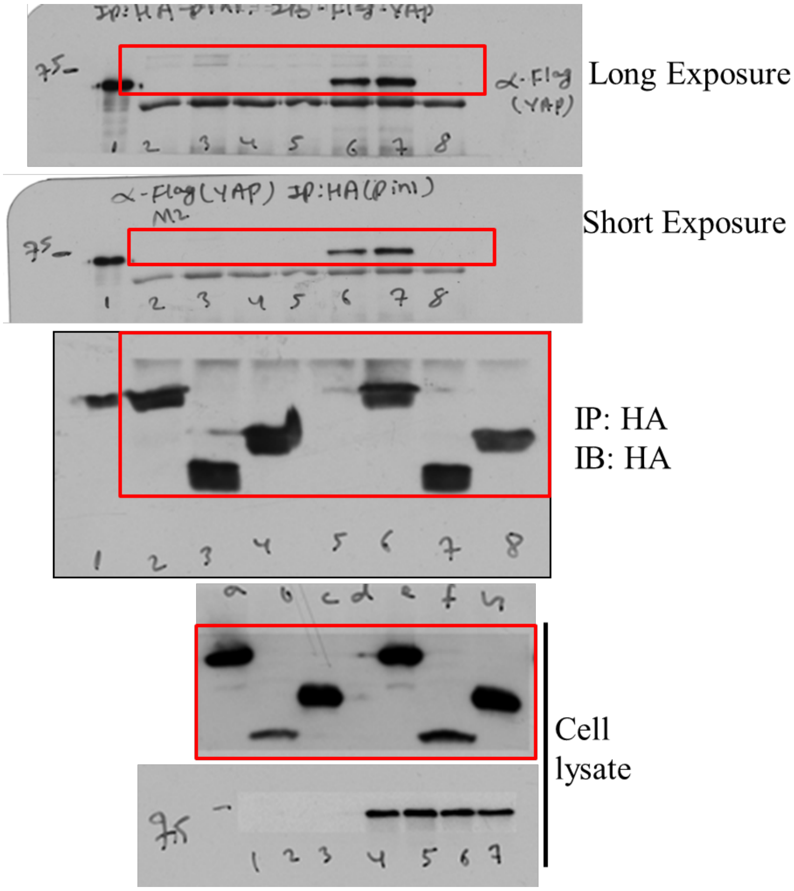
Long Exposure
Short Exposure
IP: HA
IB: HA
Cell lysate

(E)

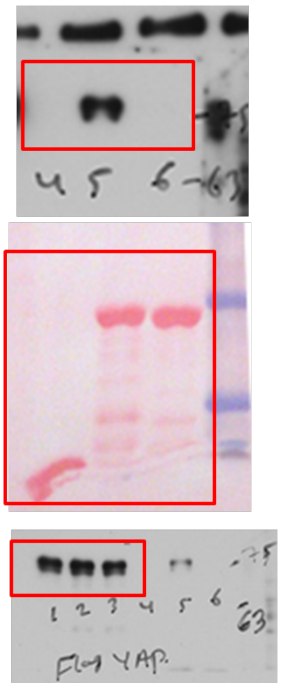

(F)

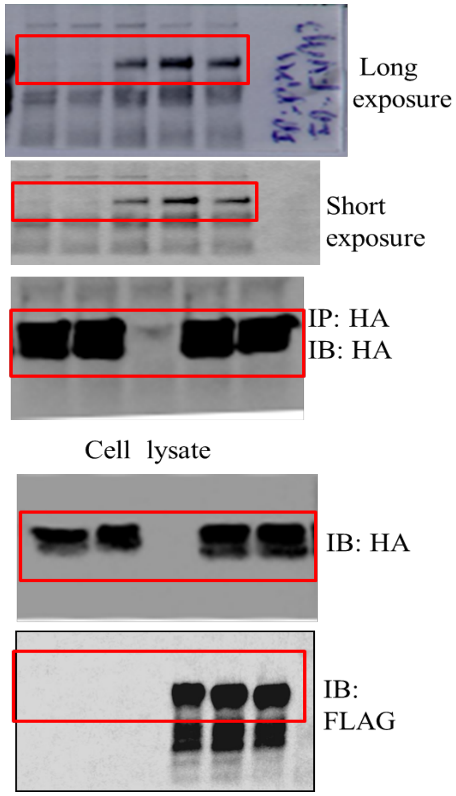

Supplementary Figure 8. Original blots used in Figure 2**.** (A) Original blots used to make Fig.2A. The bands used to make figures are highlighted with square. (B) Original blots used in Fig. 2B, the highlighted parts were used to make final figure. (C) Original blots used in Fig.2C, the portions of blots used in figures are highlighted.

(A)

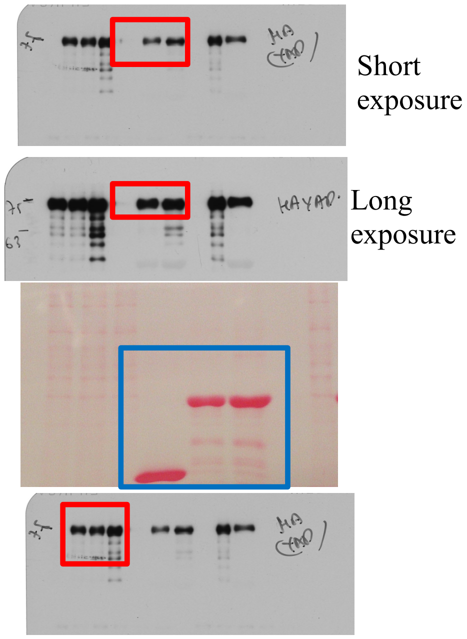

(B)

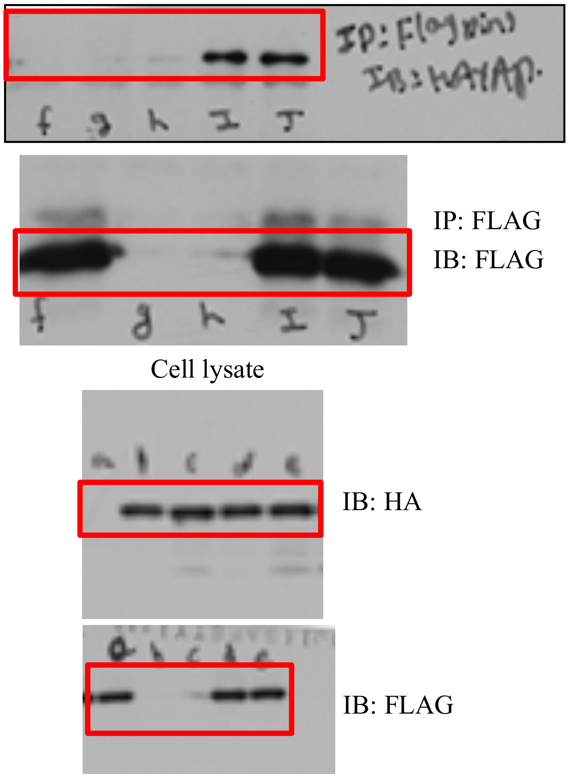

(C)

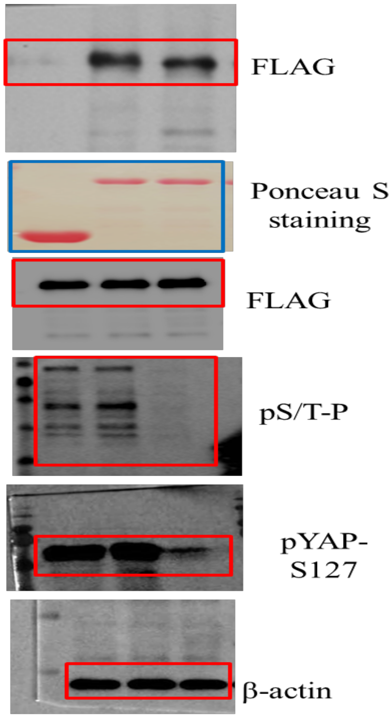

Supplementary Figure 9. Original blots used in Figure.3**.** (A) Blots used in Fig.3A, highlighted blots areas were used to make figure. (B) Original blots used in Fig.3B, highlighted areas were cropped to make figure. (C) Full blots used in Fig. 3C. (D) Full blots of cropped blots used in Fig.3D, Images were taken using Amersham Imager from GE-Health care. (E) Full blots of crop blots used in Fig.3E, the parts of blots used in figure are highlighted with in square. (F) Full blots of crop blots used in Fig.3F.

(A)

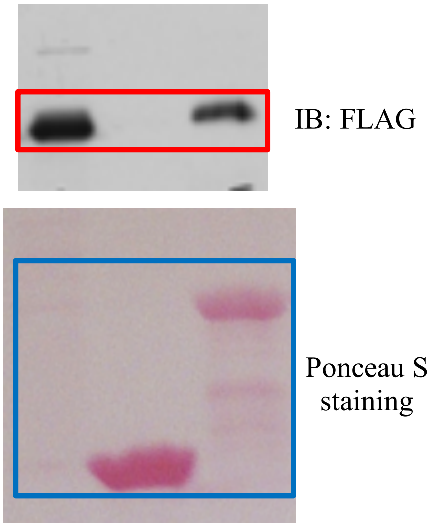

(B)

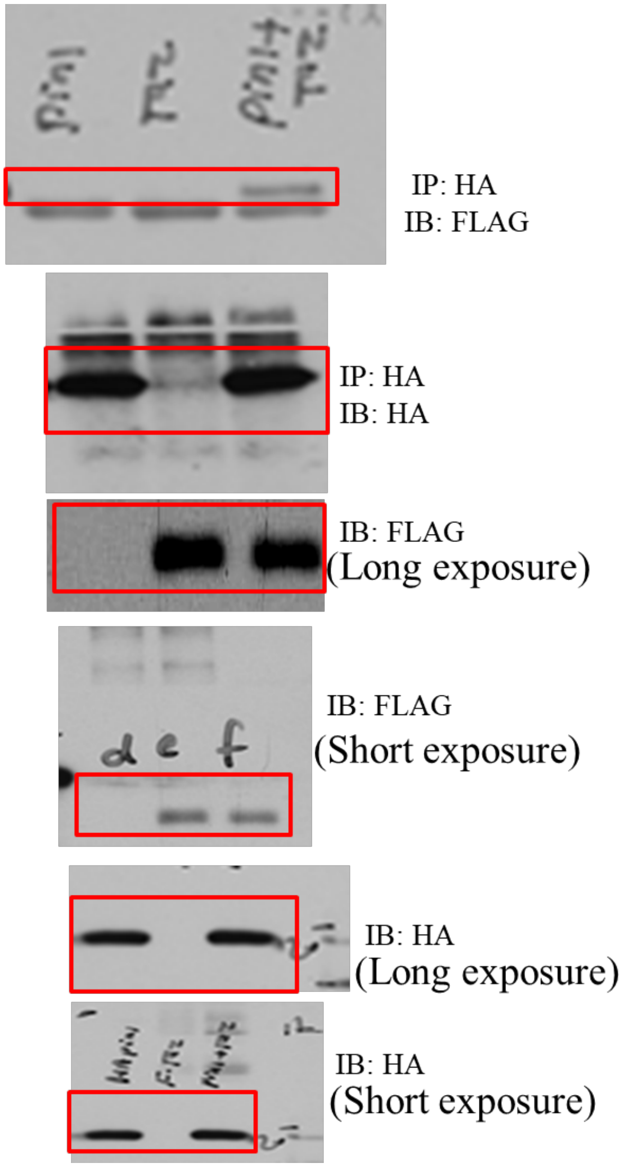

(C)

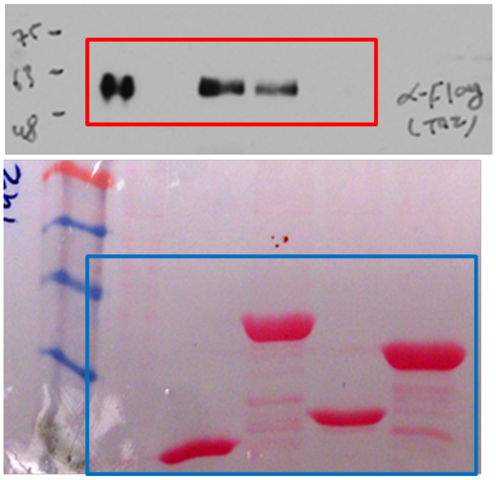

(D)

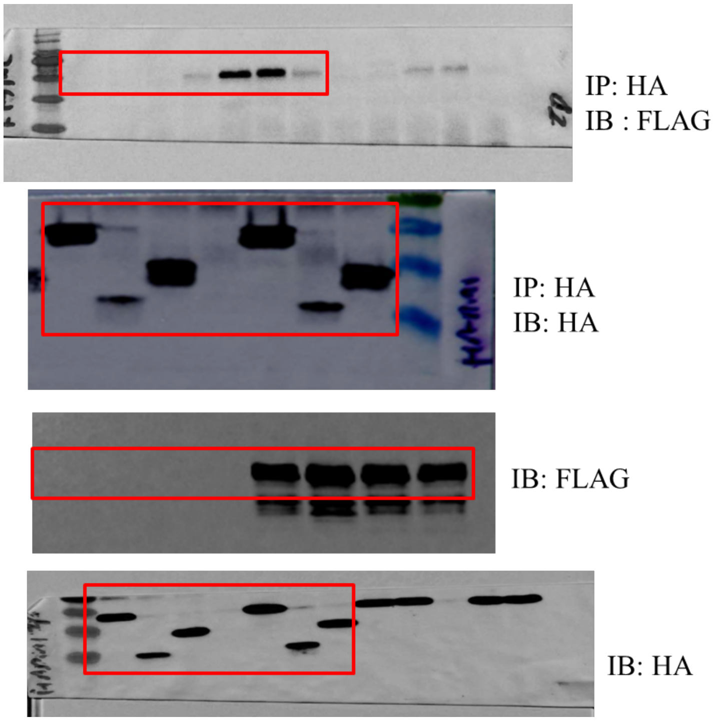
IP: HA
IB : FLAG
IP: HA
IB: HA
IB: FLAG
IB: HA

(E)

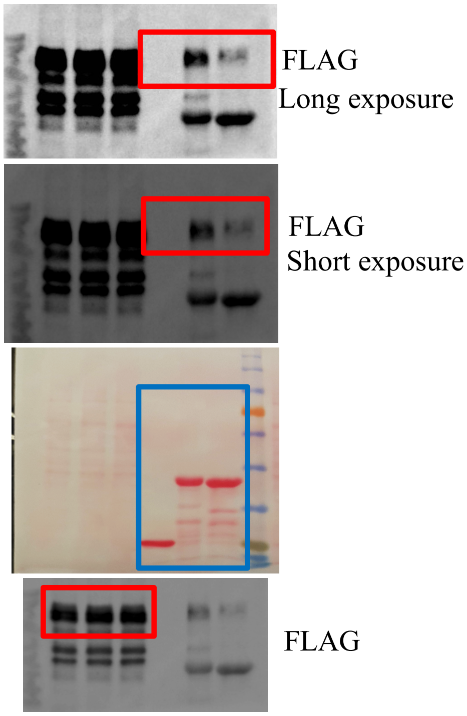
FLAG
Long exposure
FLAG
Short exposure
FLAG

(F)

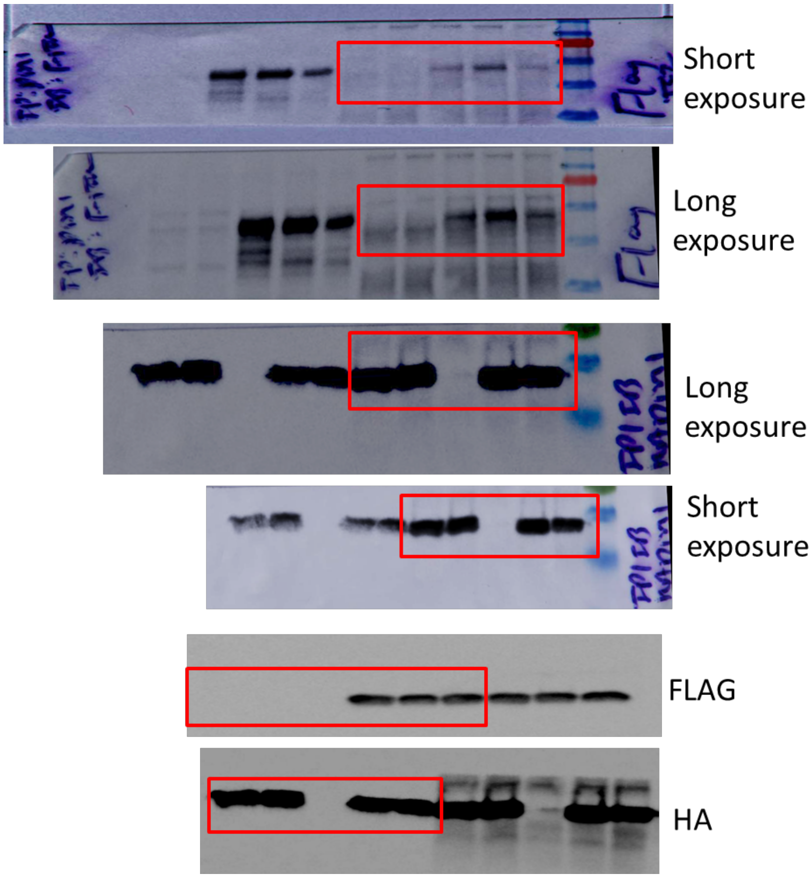
Short
exposure
Long
exposure
Long
exposure
Short
exposure
FLAG
HA

Supplementary Figure 10. (A) Full blots used in Fig. 4A, highlighted parts were used to make figure. (B) Full blots of cropped blots used in Fig. 4B. (C) Full blots of cropped blots used in Fig. 4C, highlighted parts were cropped to make figures.

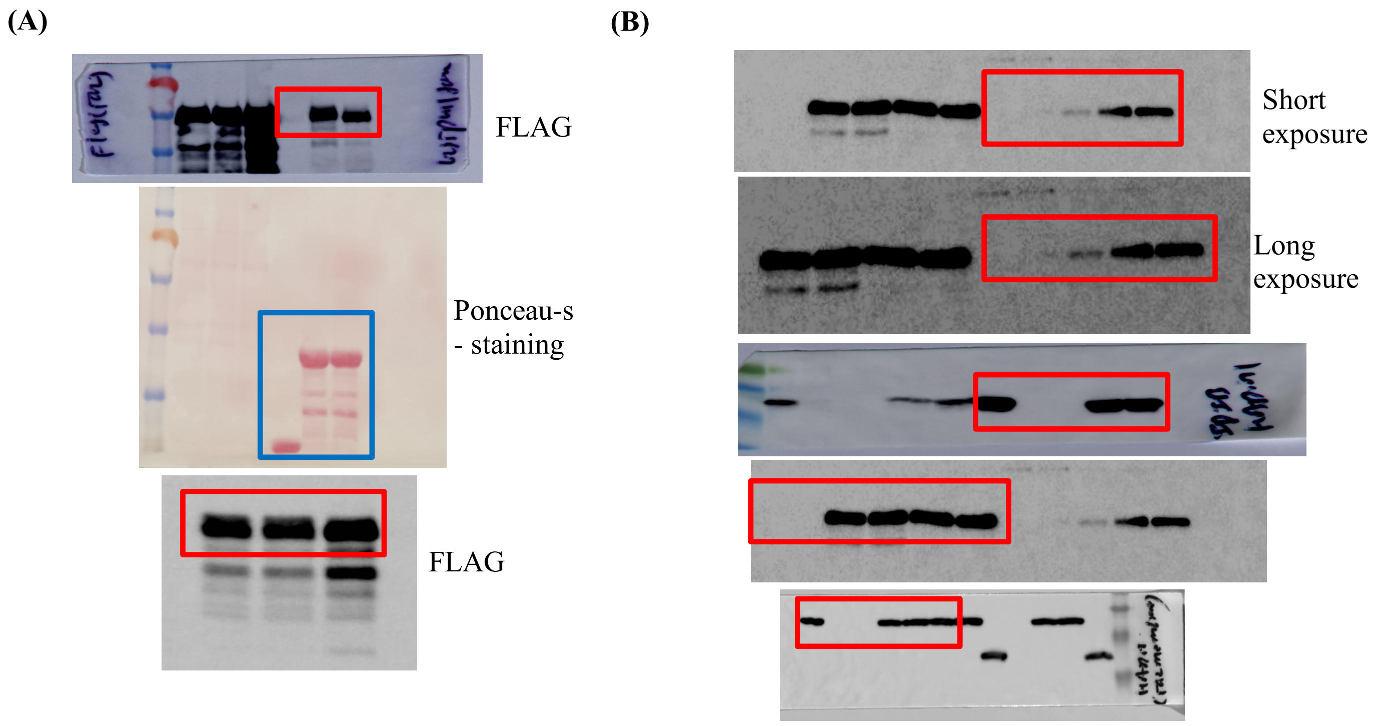

(C)

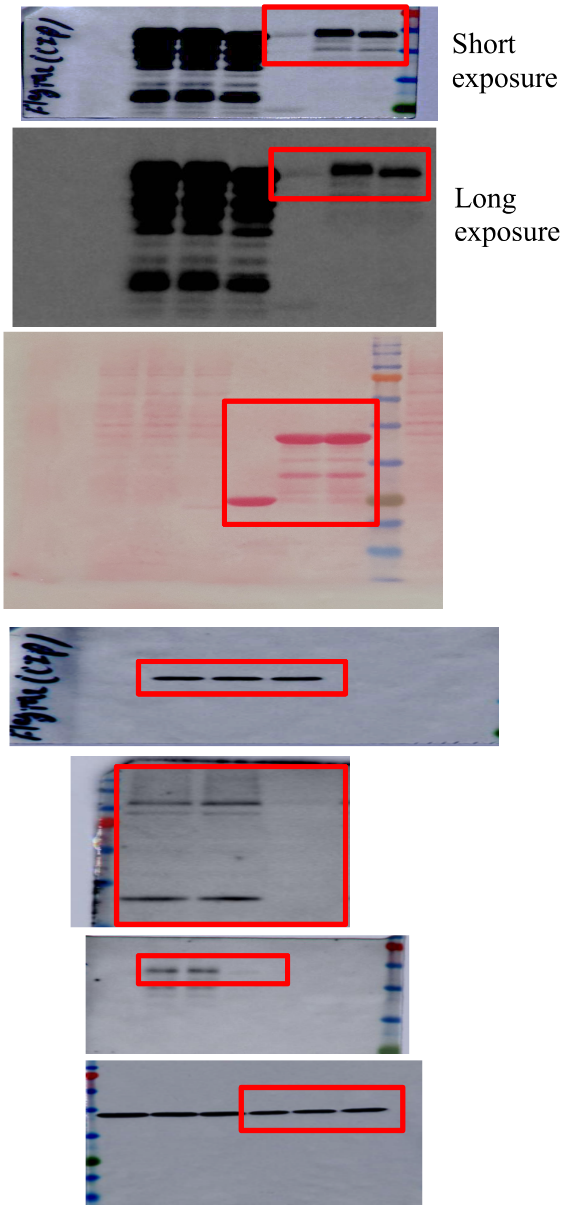

Supplementary Figure 11. (A) Full blots of cropped blots used in Fig. 5A, highlighted parts were used to make figure. (B) Original blots used to make Fig. 5B, highlighted parts were cropped to make figure. (C) Full blots of cropped blots used in Fig. 5C, highlighted parts were used to make figures.

(A)

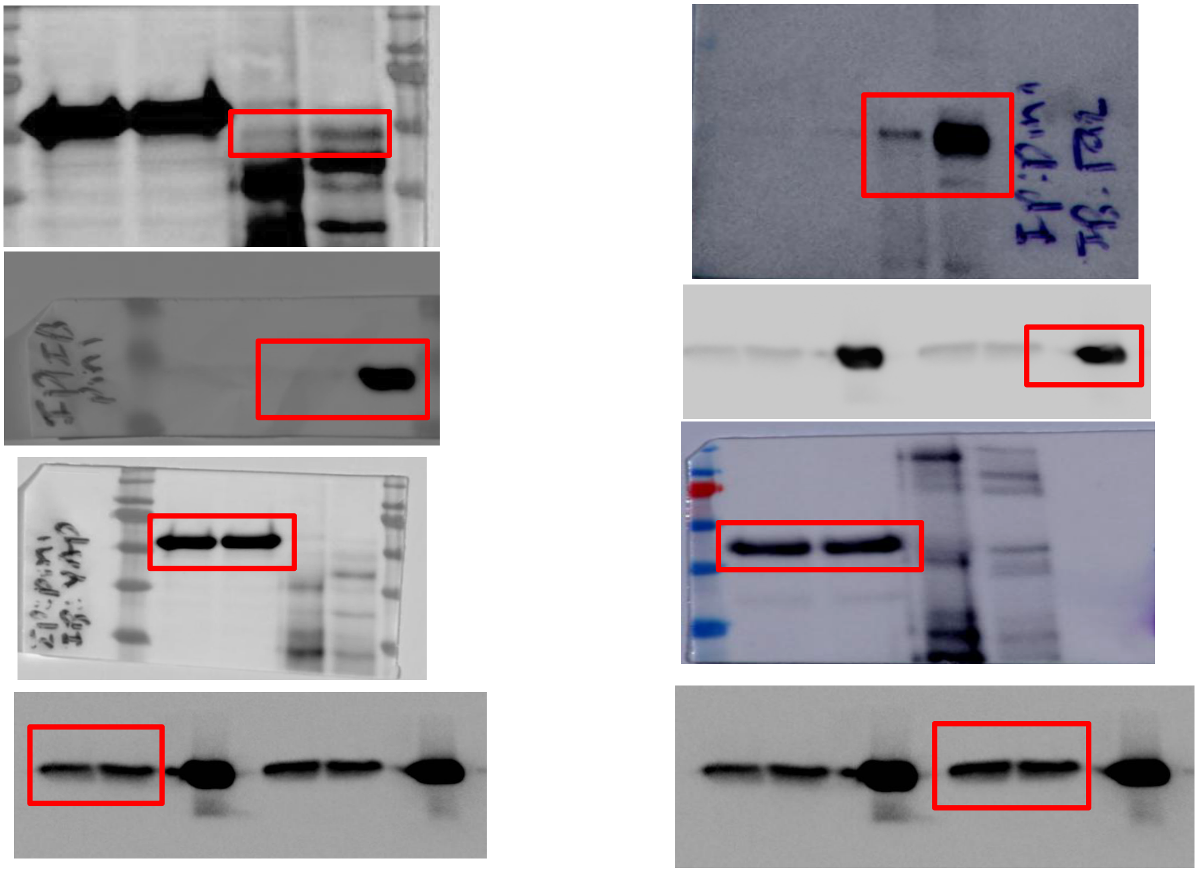

(B)

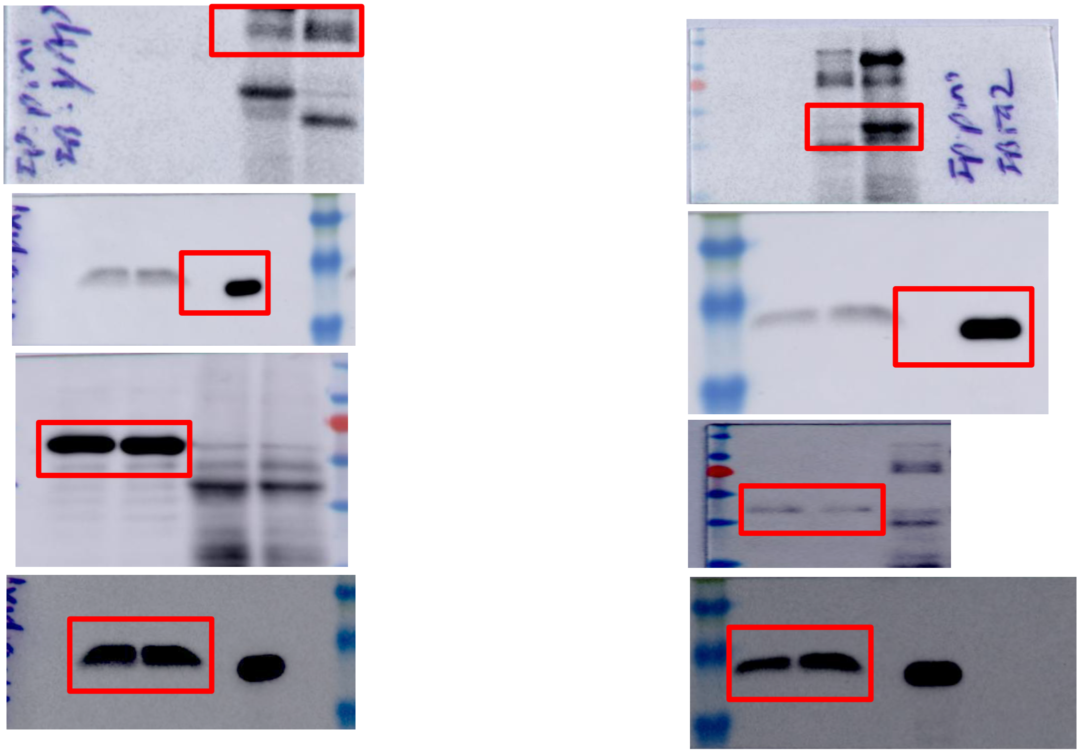

(C)

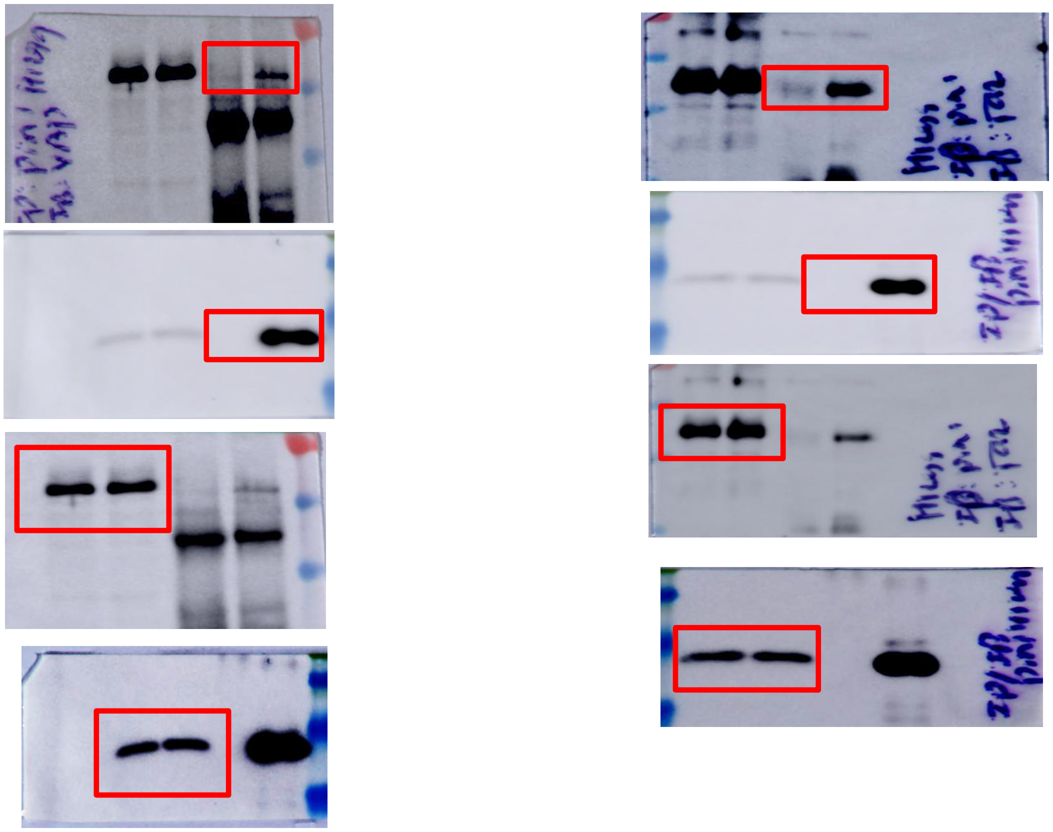

Supplementary Figure 12. (A) Full blots of cropped blots used in Fig. 6A, highlighted parts were cropped to make figures. (B) Full blots used to make Fig. 6B, cropped parts were highlighted. (C) Original blots used in Fig.6C. (D) Full blots of cropped blots used in Fig. 6D, highlighted parts were cropped to make figures.

(A)

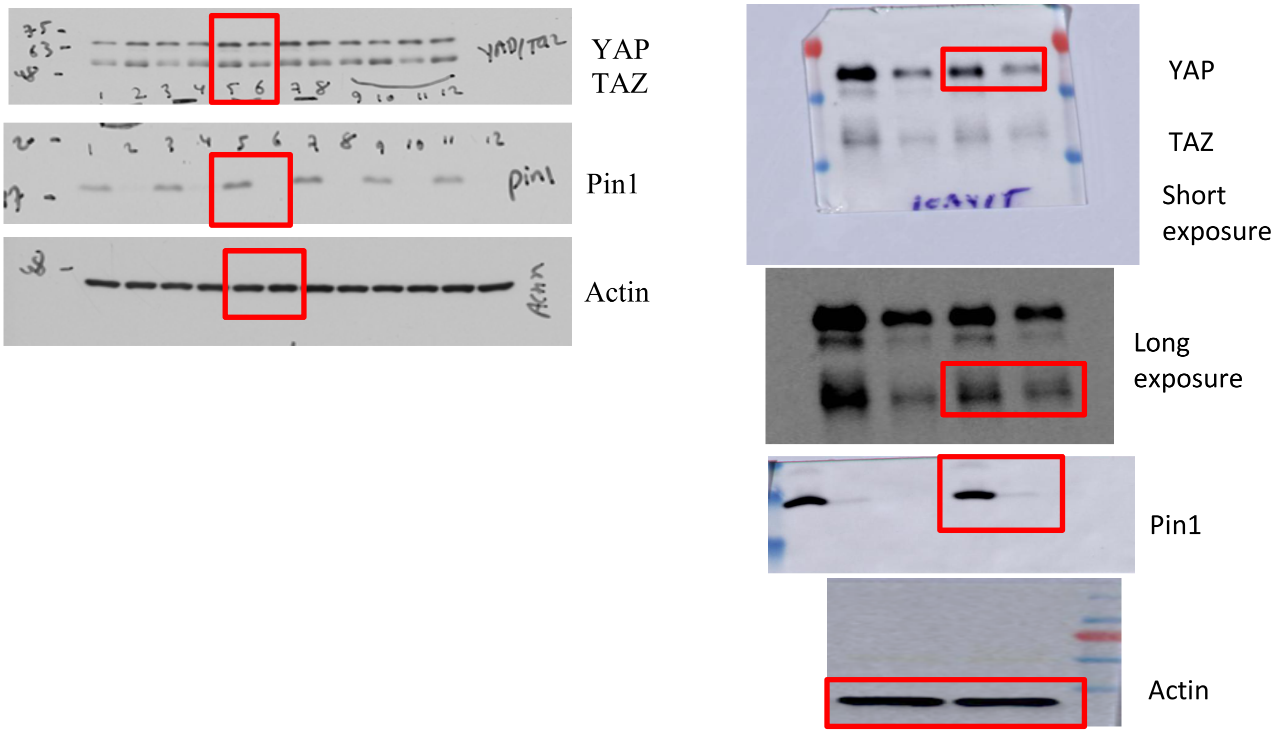

(B)

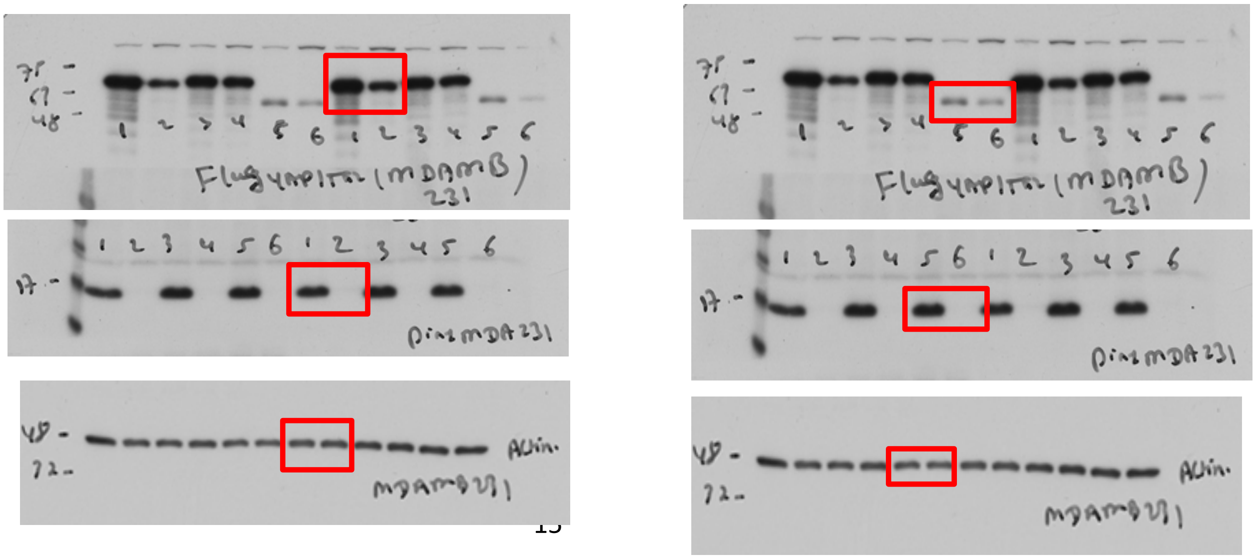

(C)

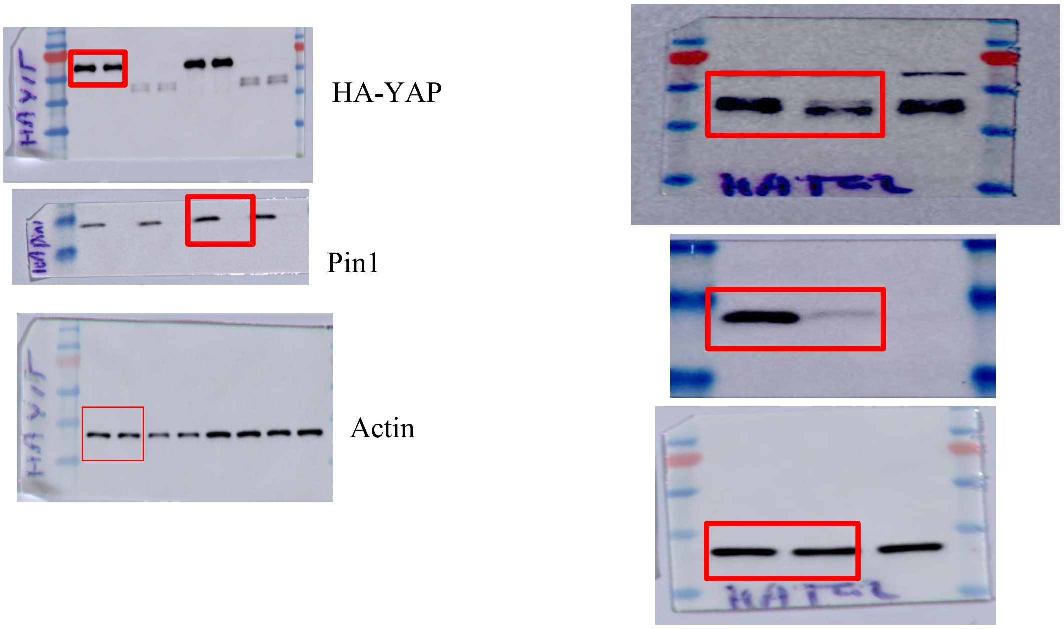

(D)

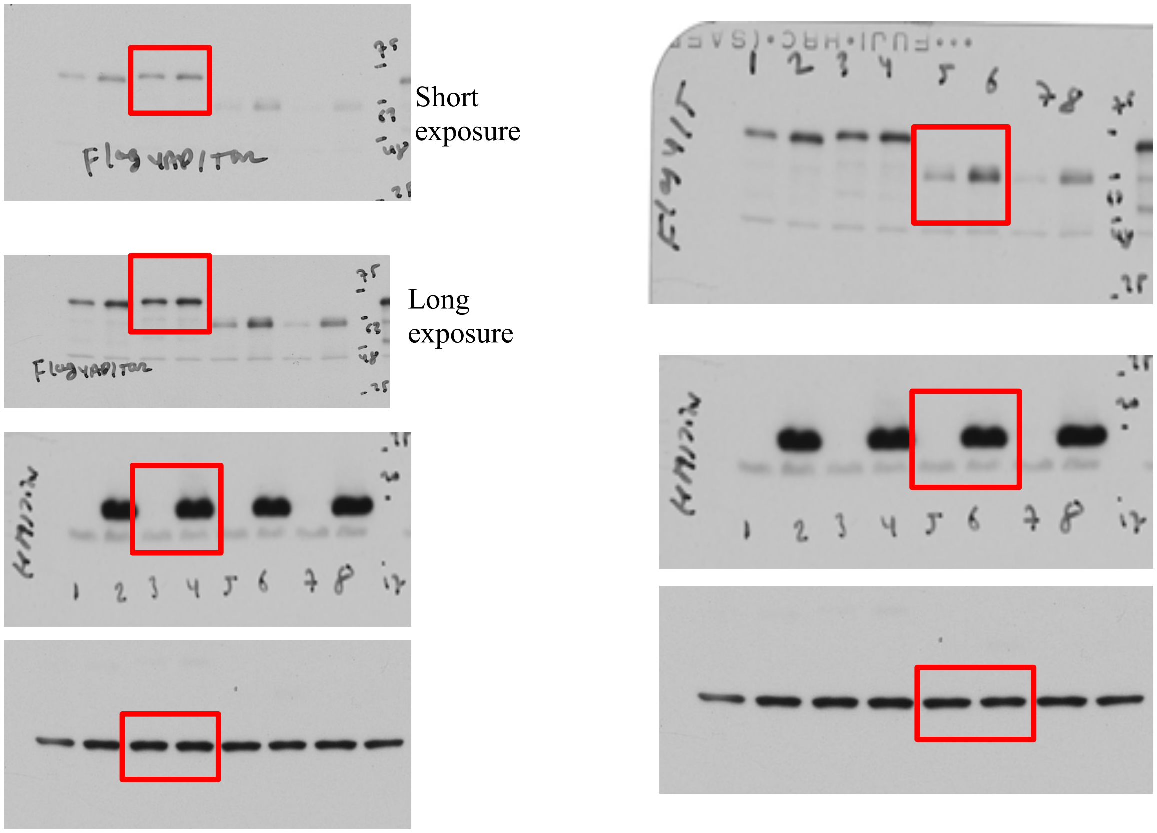

Supplementary Figure 13. (A) Full blots of cropped blots used in Fig. 7A, highlighted parts were cropped to make figures. (B) Full blots used in Fig. 7C, highlighted parts were used to make figure.

(A)

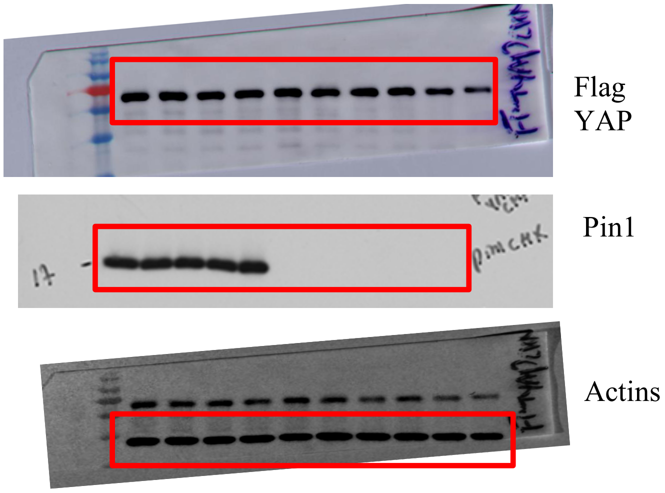

(B)

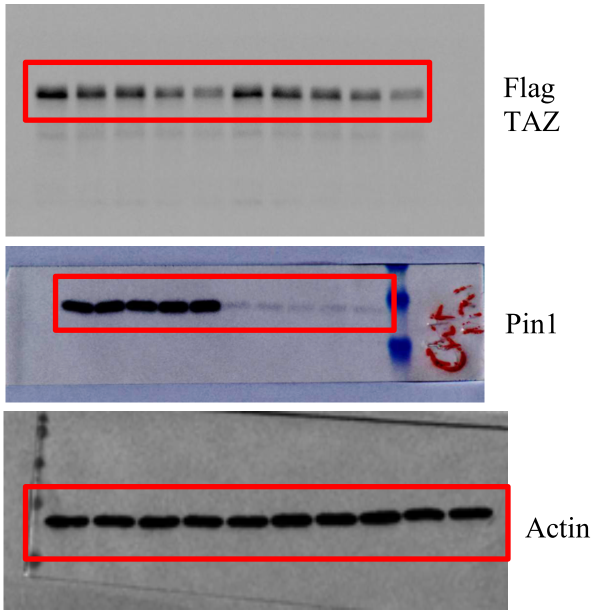

Supplementary Figure 14. (A) Full blots of cropped blots used in supplementary Fig. 2A, highlighted parts were cropped to make figures. (B) Full blots of cropped blots used in supplementary Fig. 2B.igures.

(A)

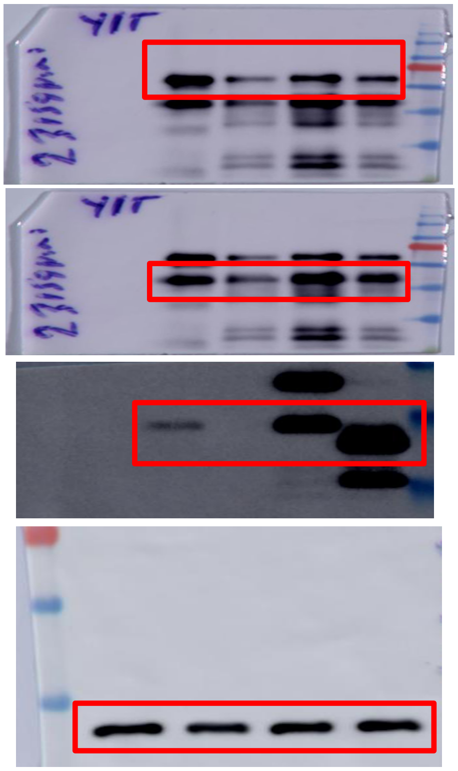

(B)

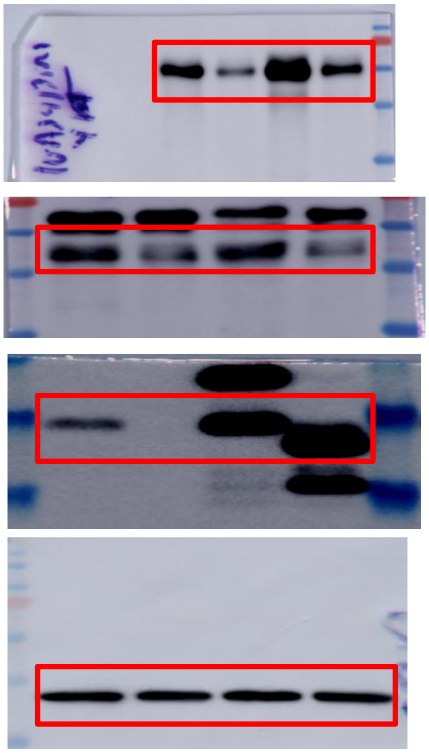

Supplementary Figure 15. (A) Full blots of cropped blots used in supplementary Fig. 3A, highlighted parts were cropped to make figures. (B) Full blots of cropped blots used in supplementary Fig. 3B.igures.

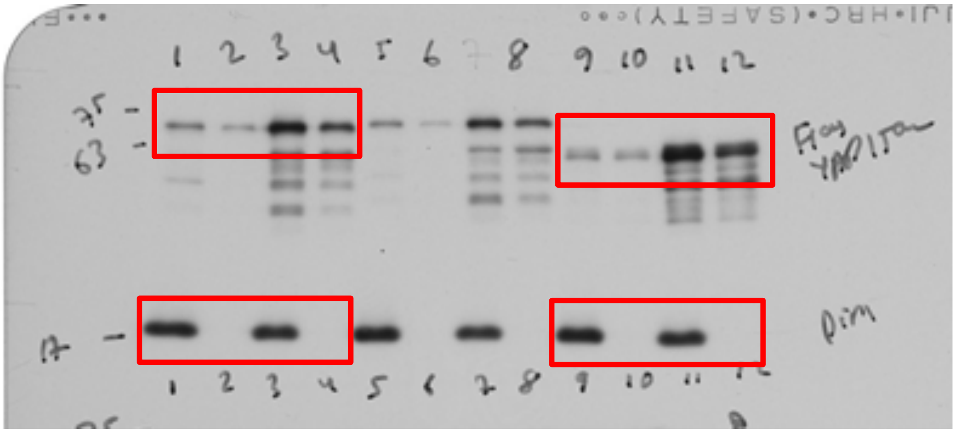

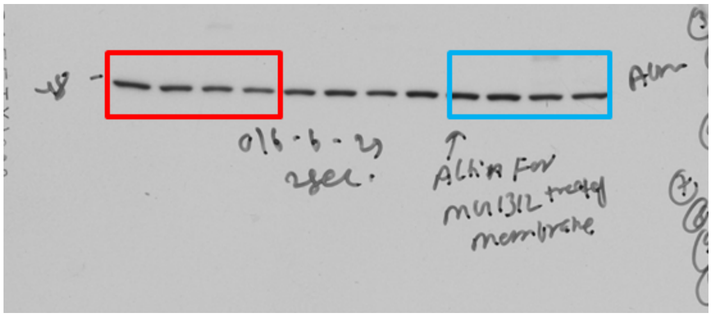

Supplementary Figure 16. (A) Full blots of cropped blots used in supplementary Fig. 6A, highlighted parts were cropped to make figures. (B) Full blots of cropped blots used in supplementary Fig. 6B.igures.

(A)

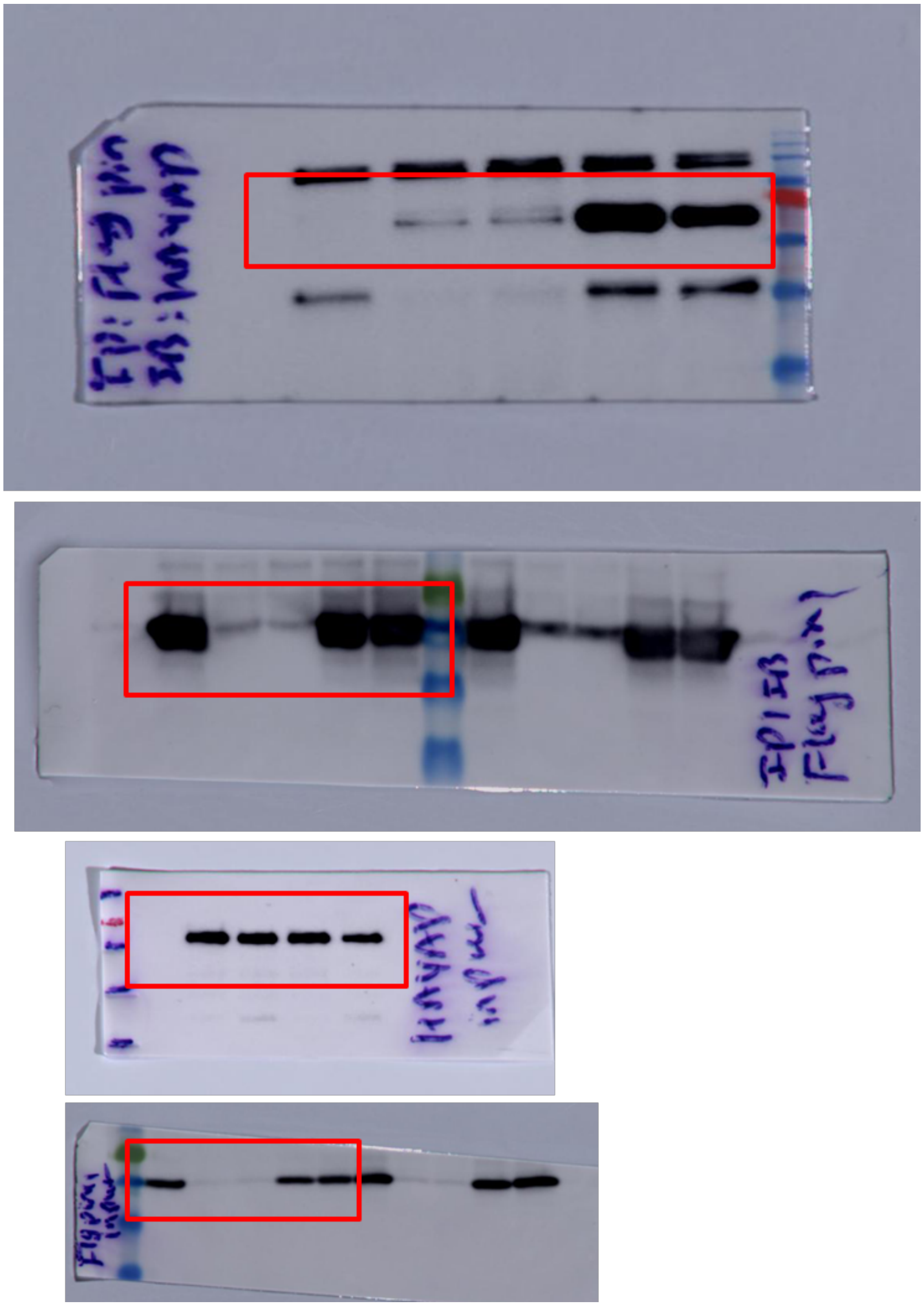

(B)

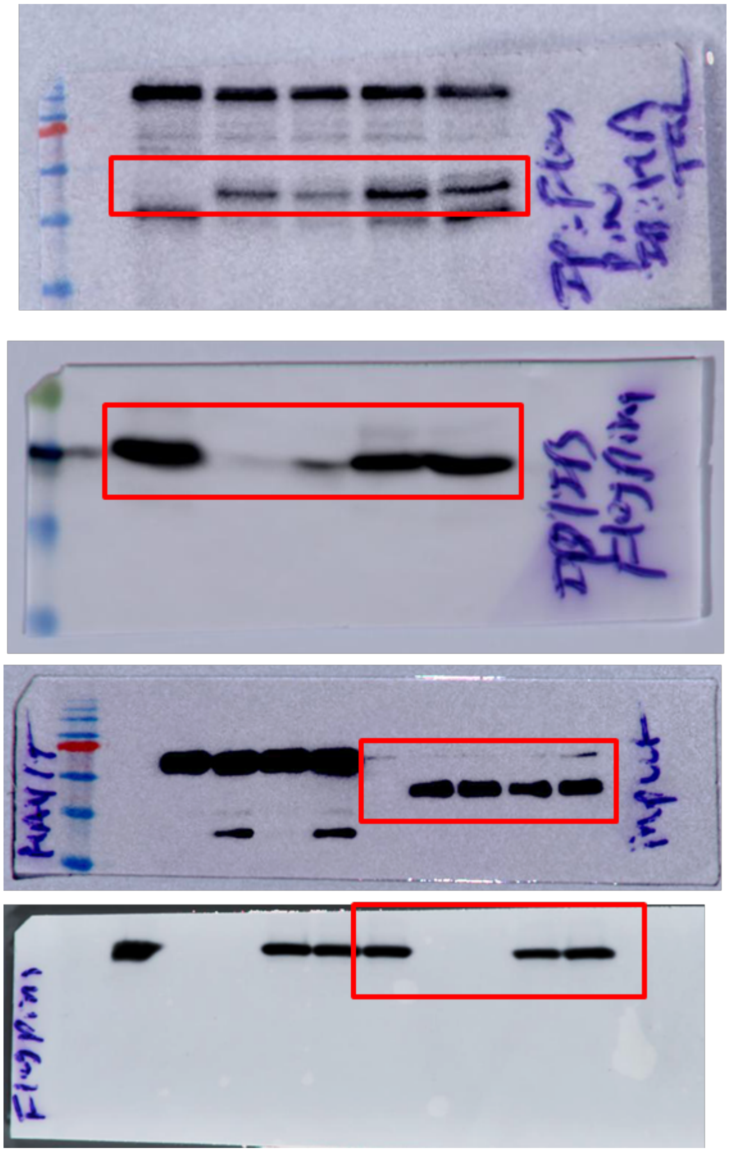

Supplement: Supplementary file 1 — Supplementary information [file 41598_2019_42767_MOESM1_ESM.pdf]
